# Feeding state-specific hormonal tuning of neural circuit modulation

**DOI:** 10.1152/jn.00164.2025

**Published:** 2025-07-14

**Authors:** Logan J. Fickling, Aaron P. Cook, Wenxin Wu, Angel Erbey Ibarra, Lingjun Li, Michael P. Nusbaum

**Affiliations:** 1 Department of Neuroscience, Perelman School of Medicine, University of Pennsylvania, Philadelphia, Pennsylvania, United States;; 2 Department of Chemistry, University of Wisconsin-Madison, Madison, Wisconsin, United States;; 3 School of Pharmacy, University of Wisconsin-Madison, Madison, Wisconsin, United States

**Keywords:** central pattern generator, gastric mill rhythm, mass spectrometry, neuropeptide, stomatogastric ganglion

## Abstract

Studies of hormone influences on neural circuits and behavior have primarily focused on the manipulation of individual hormones. Here, we examine the influence of behavioral (feeding) state-specific hormonal environments on the gastric mill (chewing) circuit configured by the neuropeptide Gly^1^-SIFamide (G-SIFamide) in the isolated *Cancer borealis* stomatogastric ganglion (STG). The G-SIFamide-activated gastric mill rhythm, which is similar to that driven by the G-SIFamidergic projection neuron modulatory commissural neuron 5 (MCN5), is distinguished from other gastric mill rhythms by the presence of rhythmic, prolonged inferior cardiac (IC) neuron bursts and associated interruptions of the pyloric rhythm. Applying 1 μM G-SIFamide in saline only occasionally activated the gastric mill rhythm, whereas this rhythm occurred more frequently when 1 μM G-SIFamide was applied in hemolymph from an unfed crab and even more often in 1-h postfed hemolymph. No novel gastric mill neuron activity occurred under these latter conditions, suggesting that hemolymph strengthened the G-SIFamide actions. Supporting this suggestion, 10 μM G-SIFamide in saline elicited this rhythm as frequently as 1 μM G-SIFamide in unfed hemolymph. Moreover, any G-SIFamide application following an initial application of 1 μM G-SIFamide in hemolymph (unfed or fed) or 10 μM G-SIFamide in saline, but not 1 μM G-SIFamide in saline, activated the gastric mill rhythm less frequently. Mass spectrometry analysis indicated that the hemolymph influence was unlikely due to additional G-SIFamide, because no SIFamide peptide family members were identified in either hemolymph. These results suggest that one or more non-SIFamide hormones strengthen this neuropeptide-modulated circuit output by increasing the effectiveness of the applied G-SIFamide.

## NEW & NOTEWORTHY

We establish that a complete, natural hormonal environment (hemolymph) increases the likelihood of a neuropeptide activating the gastric mill (chewing) rhythm in the crab stomatogastric ganglion (STG). The similar action of a higher neuropeptide concentration in saline, its comparable desensitizing effect to that of neuropeptide plus hemolymph on subsequent neuropeptide applications, and the absence of that neuropeptide in hemolymph suggest one or more distinct hormones act to enhance the effectiveness of the applied peptide.

## INTRODUCTION

The impact of individual hormones on neural circuits and behavior has been extensively studied (1–15). However, a multitude of hormones co-circulate, and the composition of these populations can change with behavioral state (8, 10, 15–18). The presence of co-circulating hormones provides the possibility for parallel modulation of neurons and circuits, and hence behavior. Thus far, however, few studies have examined the response of well-defined neural circuits to a naturally occurring, complete hormonal environment from any particular behavioral state (19–22).

It is advantageous to examine how well-defined circuits respond to hormonal modulation. This is because neural circuits are multifunctional constructs whose component neurons can change their cellular and/or synaptic properties under different behavioral states/modulatory conditions, resulting in different circuit states that can respond differently to an unchanged influence (23–29). Thus, elucidating the hormonal influence on a neural circuit is optimized by focusing on a specific circuit state, in addition to a defined behavioral state.

One system where these conditions are met is the isolated stomatogastric nervous system of the crab *Cancer borealis*, which contains the gastric mill (chewing) and pyloric (passage of chewed food) circuits in the stomatogastric ganglion (STG) (25, 30–34). Most *C. borealis* STG neurons (22 of 26 neurons) participate in the gastric mill and/or pyloric rhythms (35). The complete stomatogastric nervous system also includes the paired commissural ganglia (CoGs) and unpaired oesophageal ganglion (OG). Nearly all projection neurons that innervate the STG and influence gastric mill and pyloric circuit activity are located in the CoGs (30, 36).

The gastric mill rhythm is a relatively slow (cycle period ~10–20 s), episodic motor pattern whose circuit neurons control chewing by eliciting rhythmic muscle contractions that cause the teeth, which are located in the middle stomach compartment, to alternately protract and retract (37–39). The pyloric rhythm is a faster (cycle period ~1 s), continually active motor pattern responsible for the filtering and passage of chewed food through the posterior stomach compartment to the midgut (33, 40). Both of these circuits, which are composed of overlapping sets of STG neurons, are configured into different circuit states when influenced by different modulatory neurons or applied neuromodulators, including individual hormones (9, 24, 26, 32, 34, 41–47).

The influence of behavioral state-specific hormone populations can be studied in the isolated stomatogastric nervous system by applying hormone-transporting hemolymph directly to the STG (19). This approach is a reasonable model for the in vivo condition because the STG is located within a major artery and is continually perfused with hemolymph containing co-circulating hormones (16, 48). Moreover, the peptide hormone composition is distinct in hemolymph from crabs not fed for ≥24 h (unfed hemolymph, UH) versus hemolymph from crabs fed 1 h previously (fed hemolymph, FH) (16). Fed, but not unfed, hemolymph does influence the gastric mill and pyloric circuit states configured by stimulating the mechanosensory ventral cardiac neurons (VCNs) in preparations containing the complete stomatogastric nervous system (19). VCN stimulation influences the STG circuits via its lasting activation of two CoG projection neurons [modulatory commissural neuron 1 (MCN1), commissural projection neuron 2 (CPN2)] (49).

To determine whether hemolymph has a comparable influence on a different gastric mill circuit state, we elicited the gastric mill rhythm by incubating the isolated STG with the neuropeptide Gly^1^-SIFamide [G-SIFamide: GYRKPPFNGSIFamide; 1381.74 Da] at 1 μM, in saline or hemolymph. This peptide concentration is submaximal for activating the gastric mill rhythm (50), enabling us to identify changes in either direction regarding the likelihood of gastric mill rhythm activation. The G-SIFamide-activated gastric mill rhythm is distinct from other gastric mill rhythms in *C. borealis*, such as those activated by the VCN, post oesophageal commissure (POC), or MCN1 neurons or bath-applied CabPK peptide (19, 26, 29, 30, 49) in that it features long duration [>1 s vs. ~0.25 s] inferior cardiac (IC) neuron bursts and concomitant interruptions of the pyloric rhythm (50, 51). When dissolved in saline and superfused across the isolated STG, the most common effect of this G-SIFamide concentration was activation of a gastric mill rhythm-related pattern, which occurred in ~40% of preparations (50). This pattern was often limited to coordinated rhythmic bursting of the lateral teeth subsystem.

We performed peptide incubations instead of superfusions to accommodate the small volume of hemolymph obtained from each crab. STG incubations with G-SIFamide (1 μM) in saline only sometimes activated a complete gastric mill rhythm, whereas the frequency of this occurrence increased considerably during incubations with 1 μM G-SIFamide in hemolymph from unfed or fed crabs, with fed hemolymph being more effective. The increased frequency of occurrence of this gastric mill rhythm with 1 μM G-SIFamide in unfed hemolymph was comparable with applying a 10-fold higher peptide concentration (10 μM) in saline. In addition, 1 μM G-SIFamide in either unfed or fed hemolymph, and 10 μM G-SIFamide in saline, caused a comparably desensitized gastric mill circuit response to subsequent G-SIFamide incubations. Finally, mass spectrometry analysis of both hemolymph states revealed no detectable SIFamide peptide family members.

These results support the hypothesis that one or more non-SIFamide hemolymph hormones increase the likelihood of gastric mill rhythm generation by 1 μM G-SIFamide in a manner comparable with applying a higher G-SIFamide concentration. In addition, the greater effectiveness of fed hemolymph suggests that it contains a higher concentration of the relevant hormone(s) and/or a lower concentration of antagonistic (i.e., inhibitory) hormones.

## MATERIALS AND METHODS

### Animals

**Figure 1.**
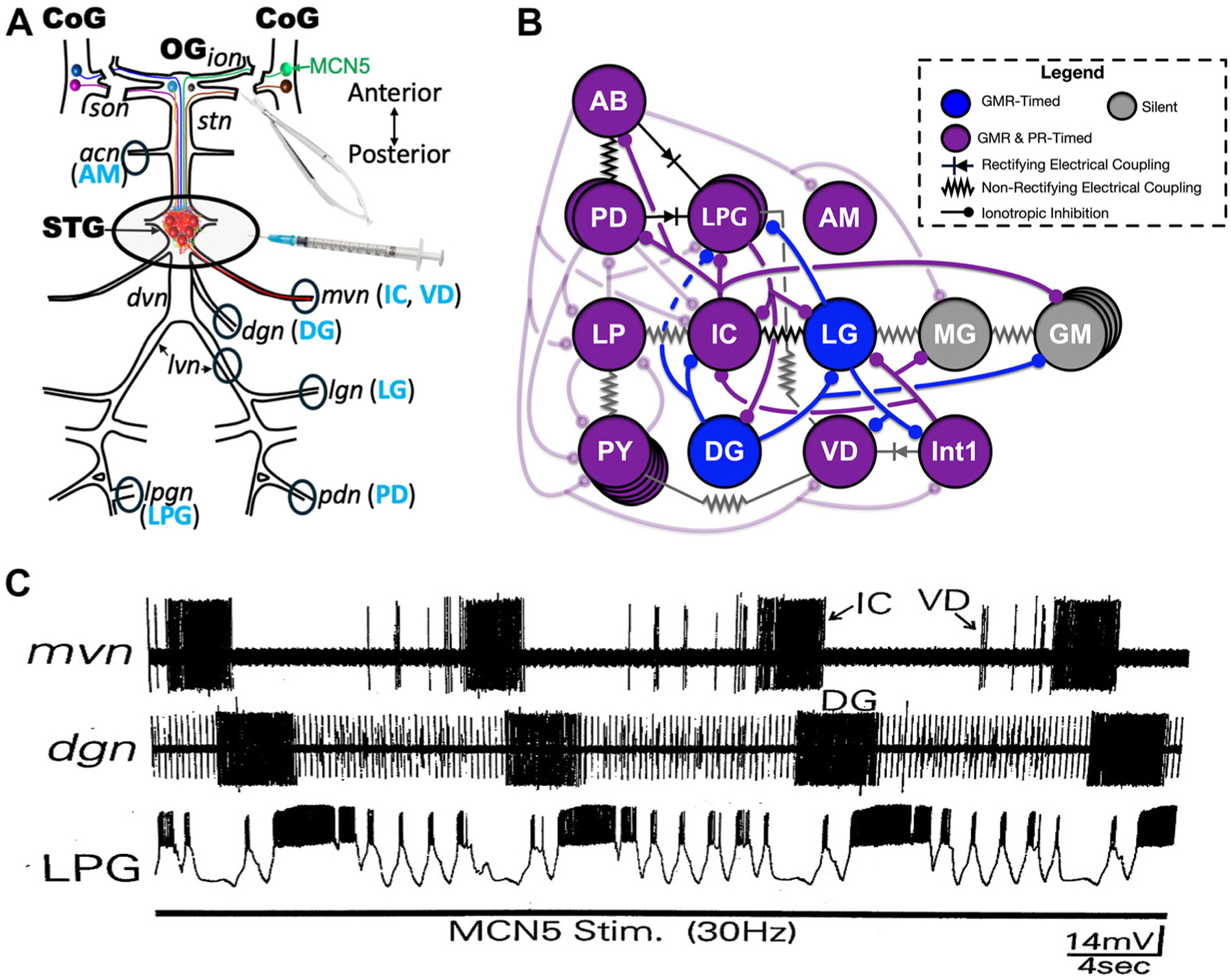
The isolated stomatogastric nervous system of the crab *Cancer borealis*, including the G-SIFamide modulated stomatogastric ganglion (STG) circuit schematic and the gastric mill rhythm driven by the G-SIFamidergic projection neuron MCN5. *A*: schematic of the isolated stomatogastric nervous system, including its four ganglia [STG, oesophageal ganglion (OG), commissural ganglia (CoGs)] plus their connecting nerves and some peripheral nerves. For all experiments in this paper, the CoGs are removed by bisecting the superior oesophageal nerves (*sons*) and inferior oesophageal nerves (*ions*). Also shown is the axon projection path of the inferior cardiac (IC) neuron from the STG through the right medial ventricular n. (*mvn*) (red); IC also projects through the left *mvn*. All STG motor neurons display bilaterally symmetric axon branching patterns. The black sphere surrounding the STG represents a Vaseline barrier, enabling the STG to be superfused/incubated separately from the rest of the system. The syringe represents manual application of saline or hemolymph after superfusion is periodically terminated. Circles surrounding individual nerves represent sites of extracellular recordings. Neuron acronyms adjacent to individual nerves indicate neurons whose axons project through those nerves. Ganglia: CoG, commissural ganglion; OG, oesophageal ganglion; STG, stomatogastric ganglion. Nerves: *acn*, anterior cardiac nerve; *dvn*, dorsal ventricular n.; *dgn*, dorsal gastric n.; *ion*, inferior oesophageal n.; *lgn*, lateral gastric n.; *lpgn*, lateral posterior gastric n.; *lvn*, lateral ventricular n.; *mvn*, medial ventricular n.; *pdn*, pyloric dilator n.; *son*, superior oesophageal n.; *stn*, stomatogastric n. Neuron: AM, anterior median; DG, dorsal gastric; IC, inferior cardiac; LG, lateral gastric; LPG, lateral posterior gastric; MCN5, modulatory commissural neuron 5; PD, pyloric dilator; VD, ventricular dilator. *B*: the G-SIFamide modulated STG connectome. Colors represent the gastric mill and pyloric rhythm activity pattern(s) of each neuron during G-SIFamide modulation. *C*: example recording of the MCN5-stimulated gastric mill rhythm. Note the repeating IC, DG, LPG bursting pattern, including prolonged IC bursts and associated interruptions in the pyloric rhythm-timed LPG activity that occurs between its longer duration, gastric mill rhythm-timed bursts. The tonically active unit in the *dgn* is the muscle sensory neuron AGR, which projects through the STG to influence CoG projection neurons (52). *C* is modified, with permission, from Ref. 50.

Male Jonah crabs (*C. borealis*) were obtained from commercial suppliers (The Fresh Lobster Company, LLC; Gulf of Maine Inc.; Cape Ann Lobstermen), and maintained in aerated, filtered artificial seawater at 10–12°C. Animals dissected for electrophysiological recordings were cold-anesthetized by being packed in ice for at least 30 min before dissection. The foregut was then removed from the animal, after which the stomatogastric nervous system was dissected from the foregut in physiological saline at ~4°C and pinned down in a silicone elastomer-lined (Sylgard 184: KR Anderson) Petri dish (Fig. 1A). To improve access of hemolymph or saline to the STG, the ganglion was dorsally desheathed immediately prior to recordings. All crabs used for recordings were unfed for at least 24 h.

### Feeding

For fed hemolymph applications, crabs were first fed portions of thawed, cleaned smelt or mackerel (total mean mass: 3–5 g). Each fish was weighed prior to feeding and suspended in front of a crab using a thin, stiff wire with a slight bend at its end to prevent it from being dislodged by water currents. Feeding usually began immediately after food presentation and proceeded at an irregular but continuous pace for ~5–15 min. During the feeding process, the fish was sufficiently manipulated by the crab that the wire could be removed from the tank without disturbing the feeding process. After the entire piece of fish was ingested, the crab was briefly removed from the tank to place a zip-tie around the hindmost leg for future identification. This commenced the postfeeding interval duration. When a crab did not completely consume all visible food, it was not included in the experiment. All feeding occurred in the tanks in which the crabs were housed.

### Solutions

*Cancer borealis* physiological saline contained (in mM): 440 NaCl, 26 MgCl_2_, 13 CaCl_2_, 11 KCl, 10 Trizma base, 5 maleic acid, pH 7.4–7.6 at room temperature. All preparations were superfused continuously with *C. borealis* saline (8–12°C). To facilitate focal application of hemolymph or saline to the STG, the desheathed STG was surrounded with petroleum jelly (Vaseline; W.W. Grainger, Inc.) and superfused with normal saline (8–12°C), in parallel with superfusing the rest of the isolated stomatogastric nervous system (Fig. 1A). The solution volume of the STG compartment ranged from ~300–500 μL. G-SIFamide (MW: 1,381.74; Genscript BioTech Corp.) was stored as a 1 mM stock solution in Optima water (Fisher Scientific) and diluted immediately before use at 1 μM or 10 μM in saline or hemolymph.

### Hemolymph Incubations

*Cancer borealis* has a semiclosed circulatory system wherein several major arteries project from the heart, including one enclosing the STG (48, 53). The hemolymph empties from these arteries into a series of sinuses bathing various tissues and organs before entering veins that circulate the hemolymph back to the heart. Hemolymph was collected after first chilling the donor crab by packing in ice for ~5–15 min. An ice-chilled 25-gauge, 1.5-in. long syringe needle was then inserted into the abdomen through the arthrodial membrane at the base of the first or second pereiopod to remove two aliquots of ~750 μL hemolymph. Crabs consistently survived hemolymph extraction for at least several months. The syringes containing extracted hemolymph were stored briefly (<5 min for hemolymph alone, and <20 min for hemolymph used in conjunction with G-SIFamide) in ice while the recorded STG was prepared for hemolymph application as described later. The order of hemolymph extraction was counter-balanced for the conditions of hemolymph alone and hemolymph with G-SIFamide. To prevent potential enzymatic breakdown of G-SIFamide, we did not add the peptide until 2 min before its application in hemolymph. The donor crab was subsequently returned to the holding tank. For the saline incubations, the saline was handled equivalently to the hemolymph before its incubation.

Immediately before each saline or hemolymph incubation, the STG motor patterns were recorded for 1–5 min, during which the temperature rarely varied by >0.1°C, after which superfusion to the STG compartment was terminated and a syringe needle was used to remove a portion of the saline (~200 μL). The removed saline was replaced with the recently extracted hemolymph (or chilled saline for control incubations), using a separate chilled syringe and needle or pipette. This process was repeated twice more to ensure full replacement of the contents of the STG compartment. The STG was then incubated with the applied solution for 15 min, during which time the STG motor patterns were recorded continuously. This solution exchange process was then repeated with the recently extracted hemolymph plus peptide, or saline plus peptide. During these incubations, the temperature in the STG compartment consistently increased by ≤3°C compared with the superfusion temperature.

At the end of each saline- or hemolymph plus peptide incubation, saline superfusion was resumed and maintained for 60–90 min. Successive pairs of incubations (carrier solution alone followed by carrier solution plus peptide) were thus separated by a minimum of 1 h.

### Electrophysiology

Extracellular nerve recordings were performed using standard techniques for this system (49). Each nerve recording was obtained using a pair of stainless steel wire electrodes that included a reference electrode placed in the bath and recording electrode placed near an individual nerve and isolated from the bath by Vaseline. The tip of each wire electrode was pressed into the Sylgard that coated the dish. Each electrode pair was connected to a differential AC amplifier (Model 1700: A-M Systems), which amplified the voltage difference between the reference and recording wire. The signal was then further amplified and filtered (Model 410 amplifier: Brownlee Precision), and then acquired for playback and analysis using the Spike2 data acquisition and analysis system (Cambridge Electronic Design Ltd.). In each nerve recording, individual STG neurons were identified on the basis of their previously established axonal projections and activity patterns (39, 49). In the displayed nerve recordings, all action potentials of a given amplitude in a particular nerve are generated by the neuron indicated above a subset of those action potentials or in parentheses under the associated nerve acronym. After all recordings were established, the STG was isolated from the CoGs by bilateral transection of the inferior oesophageal nerves (*ions*) and superior oesophageal nerves (*sons*) (Fig. 1A). After its isolation, the STG was left unperturbed for at least 30 min.

### Data Analysis

Data were collected onto computer (sampling rate ~5 kHz) using Spike2. Some analyses, including cycle period, gastric mill rhythm phase-related activity for each neuron, neuron burst durations, duty cycle, number of action potentials per burst, and intraburst firing frequency were conducted on the digitized data using either a custom-written Spike2 program (“The Crab Analyzer”) or custom-written Python software (“stns,” available at: https://pypi.org/project/stns/). All other analyses were completed using the same Python software, and a repository containing all code used in the production of this paper is freely available (https://github.com/LoganJF/hormonaltuning).

Each data point in a dataset was determined by obtaining the numerical count or mean value for the analyzed parameter from consecutive gastric mill or pyloric rhythm cycles across the entire incubation period, starting 1 min after incubation onset. For analyses of hemolymph alone incubations, each data point in a dataset was determined by obtaining the mean value for the analyzed parameter (pyloric cycle frequency) from consecutive pyloric cycles across the last 5 min of incubation (no gastric mill rhythm occurred during this condition). Pyloric cycle period was defined as the duration between the onset of consecutive pyloric dilator (PD) neuron bursts. Its cycle frequency was defined as the inverse of the cycle period. The PD neurons are a component of the pyloric pacemaker group (33, 54).

Gastric mill cycle period was defined as the duration between the onset of consecutive long duration (≥1 s) IC neuron bursts. Gastric mill rhythm-related burst duration for the lateral gastric (LG) and dorsal gastric (DG) neurons, and the long (≥1 s) IC bursts, was defined as the duration between the onset of the first and last action potential within a gastric mill rhythm-related impulse burst, during which no interspike interval was longer than 2 s (approximately twice the pyloric cycle period during the gastric mill rhythm and no more than half the duration of each gastric mill phase) (55, 56). For all other neurons (i.e., gastropyloric and pyloric neurons), an interspike interval of 0.25 s was used (approximately half the duration of the fastest pyloric cycle period in our data sets). The intraburst firing rate of a neuron was defined as the number of action potentials within a burst minus one, divided by the burst duration. A long IC burst-associated pyloric rhythm interruption was defined as a duration ≥150% of the control pyloric cycle period, wherein the control pyloric cycle period was the average of all pyloric cycle periods that had <10 IC spikes between adjacent PD bursts.

### Clustering Gastric Mill Rhythm Activity

To characterize the collective activity pattern of five gastric mill rhythm-related neurons during G-SIFamide incubations, we modified a clustering analysis used previously to characterize the pyloric rhythm (57). Our analysis excluded the first 60 s of an incubation to allow for the system to attain steady-state equilibrium. For 800 s of the remaining 840 s (95%) of each incubation, we converted the four successive, nonoverlapping 200-s spike trains from recordings of the IC, PD, LG, DG, and anterior median (AM) neurons into *1*) inter-spike intervals (ISI), *2*) phase relative to the gastric mill-timed IC neuron bursting, and *3*) four additional features, including mean intraburst firing rate, the ratio of second-order to first-order ISI, the ratio between the largest and second largest ISIs for each neuron, and a “burstiness” metric calculated by $\frac{\text{max}\;\text{diff}(s)}{s_{\text{max}}}$, where *s* is a vector of sorted ISIs, and *s*_max_ is the sorted ISI for which the difference between it and the previous sorted ISI is maximum (see Ref. 57). We then converted these variable length ISI and phase vectors into a fixed length vector by calculating the deciles of these data. We *z*-scored each of these features across our dataset and then performed a principal component analysis on the top 10 components (collectively explaining >95% of our data’s total variance). We next clustered these data, based upon their similarity to other 200 s segments from our dataset (i.e., the within-cluster sum of squares is minimized), using *k*-means (*n* clusters = 3) (58).

Finally, we used a dimensionality reduction technique, t-SNE, which enabled representation of these 10-dimensional data in two-dimensional space in a manner that respected the Euclidean distance of the higher dimension (59). Although some distinct clusters exhibited minor differences, they conceptually fell into one of three categories: *1*) gastric mill rhythms (i.e., all neurons participated consistently in a coordinated pattern), *2*) irregular gastric mill rhythms (e.g., IC neuron cycle period ≥40 s and/or intermittent long IC neuron bursts) or *3*) no gastric mill rhythm.

Using this clustering approach, we determined how the gastric mill rhythm evolved across time for each peptide application, using four 200-s segments, and then compared our various G-SIFamide incubation conditions [unfed hemolymph, fed hemolymph, saline (S)] with respect to these three categories both as mean values across segments, and as dynamic changes between segments representing ~50% of our incubation duration. Unless otherwise indicated, all analyses were performed by separating our data into “gastric mill rhythms,” “irregular gastric mill rhythms,” and “no gastric mill rhythm,” determined across the 200 s nonoverlapping segments. A gastric mill rhythm was defined as consistent coordination, relative to long IC bursts, of the neurons LG, DG, and AM, plus an IC neuron-timed delay in the onset of the next pyloric rhythm cycle.

### Phase Analysis

Gastric mill rhythm-related activity within each cycle was analyzed relative to gastric mill cycles defined as extending from the onset of consecutive long IC neuron bursts. Each cycle was divided into 100 bins (i.e., *bin 0*: IC burst onset; *bin 100*: next IC burst onset). For each neuron, its action potentials (i.e., spikes) were sorted into the relevant bin for each cycle, enabling us to determine the mean no. of Spikes/Bin. Within an experiment we averaged across all cycles in each 200 s segment, and within a condition (i.e., saline alone, hemolymph alone, G-SIFamide in saline or hemolymph) we averaged across all experiments. We limited the phase analysis to segments which were clustered as gastric mill rhythms. To account for the difference in bin duration that results from differences in the cycle period that occurred across incubations both between conditions and within each condition, we normalized the data of each segment by dividing by the bin duration (no. of Spikes/Avg Bin Duration). These phase data are thus approximately equivalent to the firing rate across the gastric mill cycle.

During some G-SIFamide-activated gastric mill rhythms, the IC neuron activity pattern included a briefer gastric mill rhythm-timed burst (duration: ~1–2 s) that was followed within 5 s by a longer gastric mill rhythm-timed burst (>2 s). In such cases, we treated the briefer burst as part of the preceding cycle and began the next cycle with the longer burst. These briefer bursts were included for the calculation of some gastric mill-related parameters (e.g., burst duration, firing rate, number of bursts), but were excluded from the IC cycle period determination.

To determine significance for our phase results, we performed pair-wise comparisons across each combination of conditions, for each bin and each neuron. Due to the number of tests performed (3 conditions × 100 bins × 6 neurons = 1,800 tests), there was a high likelihood that at least some of our inferences would be erroneous (i.e., type I error—false positive outcomes) (60). To address this possibility, we *1*) used α = 0.01 as the significance threshold, *2*) only deemed a bin to be different if a condition was significantly different from the other two conditions, and *3*) only regarded as different gastric mill and pyloric neuron parameters that maintained significance for at least two consecutive bins (corresponding to ~0.25 s). Although other, more common approaches to family-wise error rate corrections exist (e.g., Bonferroni correction), we regarded our approach as balancing the tendency of these conservative approaches to reduce power toward untenable levels with the resulting failure to detect real effects (i.e., type II errors—false negative outcomes) (60, 61).

### Mass Spectrometry

A 1-mL plastic syringe with a 23-gauge needle was used to withdraw 1 mL of hemolymph via the soft joint of one of the first three pereiopods behind the claw. The collected hemolymph was immediately mixed with an equal volume of chilled acidified methanol (methanol:water:glacial acetic acid, 90:9:1, vol/vol/vol). For liquid chromatography-mass spectrometry (LC-MS) analysis, separate pooled hemolymph samples were obtained from four unfed crabs and four 1-h postfed crabs. For matrix-assisted laser desorption/ionization mass spectrometry (MALDI-MS) analysis, separate/unpooled hemolymph samples were obtained from three unfed and three 1-h postfed crabs, ensuring biological replication. Prior to neuropeptide extraction (see next paragraph), each hemolymph sample was split into two aliquots, one without and one with 5 nM G-SIFamide standard. All hemolymph withdrawals were performed at a consistent time of day, between 10:00 AM and 12:00 PM local time.

Neuropeptide extraction was conducted immediately following hemolymph collection. Samples were vortexed, bath sonicated for 5 min, and centrifuged at 16.1 rcf for 5 min. The resulting supernatant was collected, and 0.5 mL of acidified methanol was added to the remaining pellet. The pellet was then manually homogenized, followed by another round of vortexing, sonication, and centrifugation. This extraction process was repeated three times in total. The combined supernatants from all extractions were pooled and evaporated using a speed vacuum (i.e., speedvac) set to medium heat. The dried neuropeptide samples were then reconstituted in 0.1% formic acid and desalted with C18 ZipTip P10 desalting tips (MilliporeSigma) following the manufacturer instructions. The eluent was then dried via speedvac on medium heat.

For LC-MS analysis, the cleaned peptide samples were analyzed via Orbitrap Exploris 480 MS coupled to a Vanquish Neo UHPLC system (Thermo Scientific). The raw data files were analyzed using PEAKS Xpro software (Bioinformatics Solutions Inc.) via database searching against an in-house built crustacean neuropeptide database (https://www.lilabs.org/resources). For MALDI-MS analysis, 5 nM G-SIFamide standard and unfed and fed hemolymph with and without added standard were analyzed on a rapifleX MALDI-time-of-flight (TOF) instrument (Bruker Scientific, LLC). Peptide samples were reconstituted in 20 μL of 0.1% formic acid and mixed with 2,5-Dihydroxybenzoic acid (DHB) matrix (40 mg/mL) in a ratio of 1:1 and spotted on a Bruker MTP 384 polished steel target plate. Smartbeam laser was set to 90% with 2,000 shots per spot at a repetitive rate of 10^4^ Hz, and detector gain was set at the recommended voltage for the experiments after method optimization. Spectra were acquired across a mass range of 0–3,000 *m/z*.

### Figures and Statistics

Figures were produced in Python (v.3.9) with seaborn [v.0.12.2 (62)] and matplotlib [v.3.7.1 (63)]. Statistical analyses were performed using SciPy [v.1.8.1 (64)], and Pingouin [v.0.5.3 (65)]. Clustering and dimensionality reduction were performed using Scikit-learn [v.1.3.0 (66)]. Repeated-measure correlation results were determined using R (version = 4.2.4), package rmcorr (67).

For paired testing across an entire incubation period (e.g., Figs. 3 and 5), we compared either the mean parameter values (Fig. 3) or total count of occurrences (Fig. 5) across conditions. These comparisons were used to determine statistical significance using either *1*) independent-samples *t* test, *2*) Wilcoxon signed-rank test, *3*) Brunner-Munzel test, *4*) Mann–Whitney *U* test, *5*) Friedman χ^2^ test, or *6*) ANCOVA. When appropriate, we corrected for multiple comparisons with a false-discovery rate α = 0.05. For all relevant tests, we determined the effect size (i.e., the magnitude of the differences found) using the appropriate metric for each test (e.g., rank biserial correlation [RBC] for Mann–Whitney *U* test) as described in the following paragraphs. In all figures, we designate effect sizes with abbreviations of ES = S for small, ES = M for medium, and ES = L for large, using common conventions for each, whereas actual values are provided in the text and/or figure legend.

To compare the pyloric rhythm in unfed and fed hemolymph against saline incubations, we first determined the impact of the baseline speed (i.e., the pyloric cycle frequency in the preceding saline superfusion), using an ANCOVA equal slopes regression with the baseline cycle frequency as a covariate. We then compared the *y*-intercepts of saline, unfed hemolymph, and fed hemolymph via pairwise testing of group mean differences (adjusted by the covariate) using the Holm–Sidak method. Finally, we determined whether there was an effect from the incubation by taking the baseline cycle frequency of all data points in a condition, using the ANCOVA equal slopes regression line to predict the incubation result, and comparing this against a null of a line with a slope of 1 and *y*-intercept of 0 (i.e., when there is no effect of incubation) through the use of independent samples *t* test. We also compared each hemolymph incubation against a matched within-subject saline incubation, using a Wilcoxon signed-rank test and a rank-biserial correlation (*r*_rb_) to describe the magnitude of the result. Rank-biserial correlation ranges from −1 to 1, where a value of −1 indicates all values of the second sample are smaller than the first sample, and a value of 1 indicates all values of the second sample of larger than the first sample. Here, we used sign-independent values of 0.1–0.3 for small effects, 0.3–0.5 for medium effects, and 0.5–1 for large effects, based upon commonly used values in a related effect size, Spearman’s ρ (68).

To compare the total number of long (>1 s) IC bursts and no. of pyloric rhythm cycles >150% of control cycles during incubations across conditions within subject, we used a Friedman χ^2^ test and reported the effect size using Kendall’s coefficient of concordance (Kendall’s *W*), whose values ranged from 0 to 1 with larger values representing larger differences between groups. Here, small effects were defined as <0.2, medium effects ranged from 0.2–0.39, and large effects from 0.4–1 (69).

To compare 200 s segments of gastric mill rhythms obtained from our clustering results, we employed a bootstrapping procedure (10,000 iterations) whereby we randomly selected with replacement from our sample. Each iteration of random selections was the same length as the initial sample. We then used these values to construct a confidence interval [CI] using the basic method [i.e., reverse percentile] on the mean difference between conditions (70). The CI reveals both the magnitude and uncertainty of a result, whereas a *P* value establishes only the latter (71). A CI constructed from differences is deemed significant at an α level equivalent to 1 minus the confidence level (e.g., 95% [0.95] confidence corresponds to α = 0.05) (72), if the entirety of the range does not cross 0. We report all results using a 95% CI (72). When results were deemed significant at α = 0.05, we further constructed additional CI at confidence levels of 99%, 99.9%, 99.99%, and 99.999%, stopping once either α = 0.0001, or the interval crossed 0.

In addition to reporting a confidence level for some results, we report a standardized and unitless descriptor of magnitude, Cohen’s *d* (68). This metric represents differences in group means in terms of standard deviations (e.g., *d* = 1 represents 1 standard deviation difference between group means) (68). To calculate Cohen’s *d* for parameters within our 200-s segments, we first determined the mean of a parameter across all segments exhibiting a gastric mill rhythm within an experiment, then calculated Cohen’s *d* on these mean values across experiments. The Cohen’s *d* values delineate small (0.2–0.49), medium (0.5–0.79), and large (≥0.8) effect sizes (73). Unless otherwise indicated, data are presented as the means ± standard error.

For our results comparing proportions of the clustered gastric mill circuit responses to G-SIFamide across conditions (e.g., Fig. 7A; Ref. 11), we employed a χ^2^ test and describe the magnitude of the effect using Cramer’s V (68). Cramer’s V describes the magnitude/association between categorical variables, and ranges from 0 to 1 with values of 0 representing complete independence between the variables and values of 1 representing complete dependence among the variables (74). Here, we used the standard values of 0.07–0.21, 0.21–0.35, and 0.35 + for small, medium, and large effect sizes (68, 74).

To directly address if our experimental conditions (1 μM-UH, 1 μM-FH, 10 μM-S) differed in the odds of a gastric mill rhythm occurring, we first calculated the odds of this happening in each condition across all segments (no. of gastric mill rhythm segments ÷ no. of no gastric mill rhythm segments). The denominator was calculated by combining “irregular gastric mill rhythms” and “no gastric mill rhythms.” We then calculated an odds ratio between conditions (e.g., 1 μM-FH Odds ÷ 1 μM-UH Odds). An odds ratio of 1 indicates that two groups are identical. To determine significance, we constructed a 95% confidence interval around the odds ratio; significance occurred when the entirety of the interval was above or below 1, whereas confidence intervals crossing 1 were considered nonsignificant (75). Odds ratio are a unitless descriptor of magnitude (i.e., effect size), with values of 1.5, 2, and 3 delineating small, medium, and large effects (73).

To correlate the long IC bursts with the pyloric cycle periods >150% control, we used a regression. However because this was using arbitrary criteria (i.e., IC bursts >1 s, pyloric rhythm (PR) delays >150% control) requiring collapsing all events within an experiment into a single value, we additionally sought to determine the relationship across all pyloric cycles which had >20 IC spikes within a pyloric cycle (i.e., between successive PD bursts) using repeated measure correlation that estimates the common regression slope (i.e., the association shared among individuals). We reported this effect size using *r*_rm_, and *R*_rm_^2^, which respectively represent the strength of the relationship between the two variables and the proportion of variance explained in the prolonged pyloric cycle by the IC activity (67). Here, we used *r*_rm_ values of 0.2, 0.5, and 0.8 to delineate small, medium, and large effects, based upon commonly used values in a related effect size analysis with Pearson’s correlation (*r*) (73). We used *R*_rm_^2^ values of 0.02, 0.13, and 0.26 to delineate small, medium, and large effects, based upon commonly used values in related effect size from the coefficient of determination (*R*^2^) (73).

## RESULTS

The gastric mill circuit is composed primarily of motor neurons separated into two subsystems, one controlling protraction [gastric mill (GM) neurons] and retraction (DG neuron) of the dorsally tethered medial tooth and one controlling protraction [LG, medial gastric (MG), GM, IC neurons] and retraction [lateral posterior gastric (LPG), ventricular dilator (VD) neurons] of the bilaterally paired lateral teeth within the middle, gastric mill compartment of the three compartment stomach (Fig. 1B) (38). The AM motor neuron, which innervates muscles of the anterior, cardiac sac stomach compartment, also exhibits gastric mill rhythm-timed activity during some versions of the gastric mill rhythm (55, 76, 77). There is a single gastric mill interneuron (Int1), which projects to the CoGs and influences at least one STG-projecting neuron (78, 79). During most of the well-characterized gastric mill rhythms, Int1 bursts in alternation with the LG protractor neuron due to their reciprocal inhibition and thus is active during retraction (80–82). The Int1/LG pair constitutes the core rhythm generator for the aforementioned gastric mill rhythms (27, 80–82). All gastric mill neurons are present as a single copy except GM (four copies) and LPG (two copies) (Fig. 1B).

**Figure 2.**
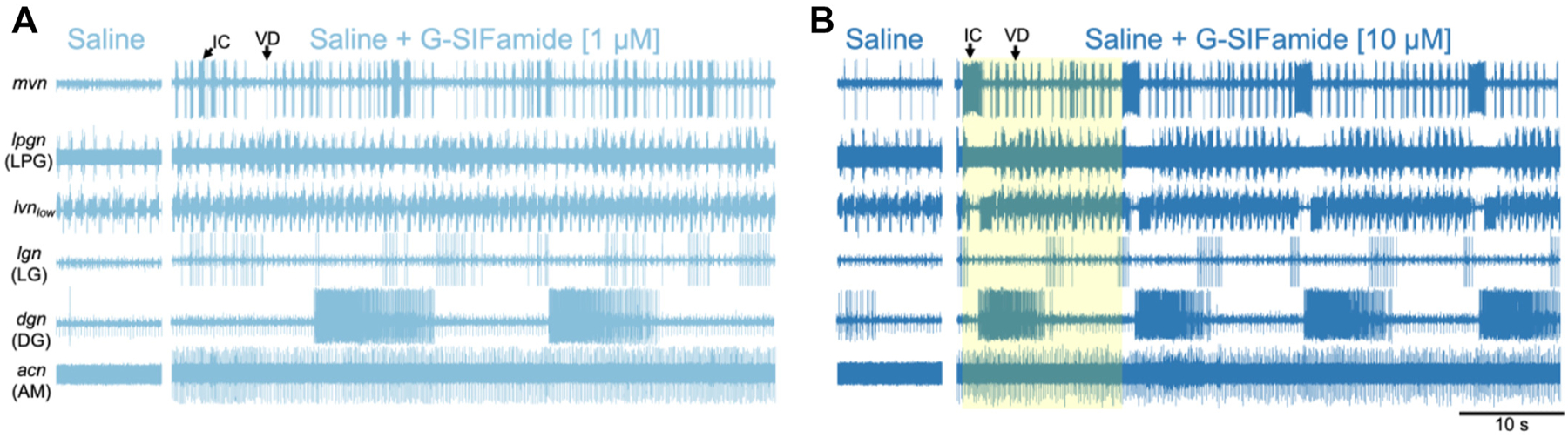
Example recordings of the gastric mill and pyloric circuit response to G-SIFamide incubation in saline with 1 μM (*A*) or 10 μM (*B*) peptide. *A*: during saline incubation (*left*), there was an ongoing pyloric rhythm [lateral ventricular n. (*lvn*)_*low*_, lateral posterior gastric n. (*lpgn*)] and no gastric mill rhythm [lateral gastric n. (*lgn*), dorsal gastric n. (*dgn*)]. Incubation with 1 μM-S (*right*) strengthened the pyloric rhythm [e.g., inferior cardiac (IC) and ventricular dilator (VD) activity; medial ventricular n. (*mvn*)] and activated the gastric mill neurons lateral gastric (LG), dorsal gastric (DG), and anterior median (AM). *B*: during saline incubation (*left*), there was an ongoing pyloric rhythm (*lvn*_*low*_, *lpgn, mvn*) and occasional DG neuron activity (*dgn*). Subsequent incubation with 10 μM-S enhanced the pyloric rhythm and activated the gastric mill rhythm, as is evident from the repeated long-duration IC bursts, associated interruptions in the pyloric rhythm, IC-coordinated bursting in the LG and DG neurons, and IC-timed increase in the AM neuron firing rate. The shaded region encompasses one gastric mill rhythm cycle.

A distinct gastric mill rhythm, activated by G-SIFamide (5 μM) superfusion, is similar to that driven by the G-SIFamidergic projection neuron MCN5 (50, 51, 83). This rhythm is readily distinguished from other gastric mill rhythms by the occurrence of prolonged gastric mill rhythm-timed IC neuron bursts and associated interruptions of the pyloric rhythm, and prolonged LPG neuron bursts that can span pyloric cycles (Fig. 1C). During the other, aforementioned gastric mill rhythms, IC and LPG exhibit only pyloric rhythm-timed bursts. As elaborated later, incubating the isolated STG with saline plus 1 μM G-SIFamide routinely enhanced STG neuron activity but only occasionally activated the gastric mill rhythm, whereas saline plus 10 μM G-SIFamide frequently activated this rhythm (Fig. 2; Supplemental Fig. S1 and Fig. S3B).

The pyloric rhythm is driven by the rhythmic bursting of the endogenous oscillator interneuron AB and its electrical coupling with the paired PD and LPG motor neurons (51, 84, 85) (Fig. 1B). During the different gastric mill rhythms, distinct but overlapping sets of gastric mill neurons also exhibit pyloric rhythm-timed activity due to synaptic inhibition from or electrical coupling with the AB neuron, or synaptic inhibition from the IC neuron, and thus exhibit a gastropyloric activity pattern (39, 80, 81, 83, 86). Because the pyloric rhythm is interrupted in gastric mill rhythm-time during the MCN5/G-SIFamide gastric mill rhythm, the pyloric neurons are also described here as exhibiting gastropyloric-timed activity (Fig. 1, B and C and Fig. 2; Supplemental Fig. S1 and Supplemental Fig. S3, A and B).

### Unfed and Fed Hemolymph Slow the Pyloric Rhythm in the Isolated STG

Selectively incubating the STG with unfed or 1-h postfed hemolymph in the complete stomatogastric nervous system (i.e., with CoGs communicating with the STG) had little impact on the pyloric rhythm relative to the saline incubations, including no change in the pyloric cycle frequency (19). However, after the CoGs are removed the pyloric rhythm slows, and sometimes stops, due to the loss of spontaneously active modulatory drive (54, 57, 87). Slow pyloric rhythms often display a cycle frequency-dependent response to modulator applications, with slower rhythms responding more vigorously than faster rhythms (9, 87–89). We therefore used an analysis of covariance (ANCOVA) equal slopes regression to quantify the proportion of variance in the pyloric cycle frequency response that is explained by the kind of incubation [i.e., saline (S), unfed hemolymph (UH), or fed hemolymph (FH)] after accounting for the covariate of the baseline cycle frequency [i.e., the saline superfusion immediately preceding the incubation].

**Figure 3.**
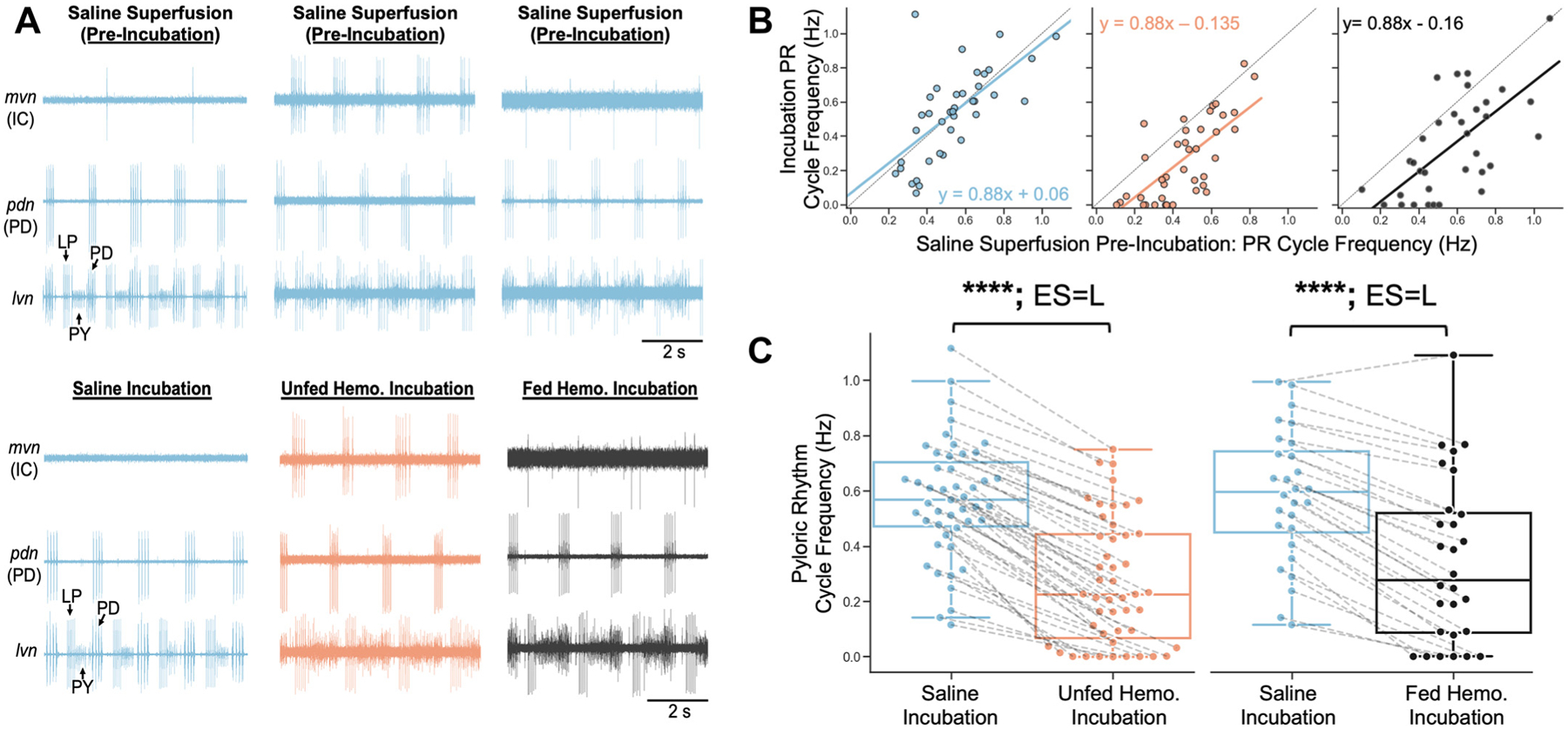
Incubating the isolated stomatogastric ganglion (STG) with unfed or fed hemolymph alone slows the pyloric rhythm. *A*: example recordings of the pyloric rhythm response in the isolated STG to incubation with saline (*left*), unfed hemolymph (*middle*), and fed hemolymph (*right*), relative to the preceding saline superfusion shown above each example. All superfusions shown display four pyloric cycles [5 pyloric dilator (PD) neuron bursts]. The distinct colors for each incubation condition match those in *B* and *C*. *B*: scatterplots of the mean pyloric rhythm (PR) cycle frequency across preparations during incubations with saline (*n* = 42) (*left*), unfed hemolymph (*n* = 47) (*middle*), and fed hemolymph (*n* = 42) (*right*), as a function of the preceding saline superfusion. The thin line is the unity line. The thicker, colored line is the equal slopes regression line, representing the best fit for each group after adjusting each group mean by the covariate of superfusion cycle frequency. Only the saline condition was not significantly different from a line with a slope of 1 and intercept of 0; note the positive *y*-intercept for saline (*b* = 0.06), but negative intercept for both unfed (*b* = −0.135) and fed (*b* = −0.16) hemolymph. *C*: box and whisker plots of the mean pyloric cycle frequency during incubations with saline vs. unfed (*n* = 47) or fed hemolymph (*n* = 28) from each preparation. Dashed lines link results from the same preparation. Bottom and top box borders represent the first (Q1) and third quartile (Q3), respectively, while the interior horizontal line represents the median value for each data set. Top and bottom exterior horizontal lines represent 1.5 × IQR (interquartile range: Q3–Q1). ****P < 0.00001, Wilcoxon signed-rank test. Saline vs. Unfed Hemolymph, Rank-biserial correlation (*r*_rb_): 0.96; Saline vs. Fed Hemolymph, *r*_rb_: 0.85. Not shown: Saline vs. Saline- n.s. (not significant), *P* = 0.55, independent samples *t* test; Unfed Hemo vs. Fed Hemo- n.s., *P* = 0.42, Brunner-Munzel test. DG, dorsal gastric; *dgn*, dorsal gastric n.; ES, effect sizes; IC, inferior cardiac; *lpgn*, lateral posterior gastric n.; *mvn*, medial ventricular n.; *pdn*, pyloric dilator n.

We found no significant interactions between the kind of incubation (e.g., saline vs. hemolymph) and the covariate of baseline pyloric cycle frequency (*P* = 0.8; ANCOVA). However, the covariate of baseline cycle frequency did correlate with the values of the incubation cycle frequency (*P* < 0.001; ANCOVA). We thus adjusted our incubation cycle frequency values by this covariate prior to group-wise comparison, which still detected differences (*P* < 0.001, ANCOVA). Subsequent pairwise testing indicated that, relative to saline incubations, there was a reduced pyloric cycle frequency response in both FH (adjusted mean difference, 0.22 Hz, *P* < 0.001, Holm–Sidak method), and UH (adjusted mean difference, 0.20 Hz, *P* < 0.001, Holm–Sidak method) (Fig. 3, A and B). In contrast, the reduction in the pyloric cycle frequency was comparable in FH and UH (adjusted mean difference, 0.02 Hz, *P* = 0.6, Holm–Sidak method) (Fig. 3B). Finally, we tested whether in each condition the regression was distinct from a null model of *y* = *x* + 0 [i.e., exhibiting no change from the preceding baseline]. We detected differences for UH (*n* = 39, *P* = 4.4 × 10^−6^, independent samples *t* test) and FH (*n* = 34, *P* = 2.9 × 10^−5^, independent samples *t* test), but detected no differences for saline (*n* = 39, *P* = 0.9, independent samples *t* test). Collectively, these results suggest that fed and unfed hemolymph incubations, but not saline incubation, had a comparable slowing effect on the pyloric rhythm (Fig. 3, A and B).

We next directly compared saline incubation against incubation with UH or FH in the same animal. As aforementioned, relative to saline, the pyloric rhythm was consistently slower in UH and FH (Cycle Freq.—S, 0.57 ± 0.03 Hz; UH, 0.27 ± 0.03 Hz, *P* = 1.1 × 10^−11^, rank-biserial correlation (r_rb_) = 0.96; S, 0.58 ± 0.04 Hz; FH 0.34 ± 0.06 Hz, *P* = 1.8 × 10^−5^, *r*_rb_ = 0.85; Wilcoxon signed-rank test), and it sometimes terminated (Fig. 3, B and C). The magnitude of this hemolymph effect was also substantial, displaying a large effect size for both unfed and fed hemolymph (Fig. 3C).

Sufficient shifts in pH can disrupt the pyloric rhythm (90), suggesting that a pH difference between *Cancer* saline and the incubation hemolymph could explain the inhibitory action of hemolymph on the pyloric rhythm in the isolated STG. To examine this possibility, we determined the pH of unfed and fed hemolymph soon after its removal from each crab. In both hemolymph types, the mean pH value was comparable with the control saline pH and their range was well within the pH values known to permit maintenance of the control pyloric cycle frequency (90) (Saline: 7.48 ± 0.02 [range: 7.4–7.6], *n* = 15; UH: 7.21 ± 0.03 [range: 6.8–7.5], *n* = 23; FH: 7.0 ± 0.03 [range: 6.9–7.4], *n* = 13). It was therefore unlikely that the hemolymph pH was responsible for the pyloric cycle frequency response to the hemolymph incubations.

### Unfed and Fed Hemolymph Enhance Gastric Mill Rhythm Activation by G-SIFamide

Superfusing G-SIFamide in saline across the desheathed STG activates or increases the activity of the gastric mill circuit neurons, including occasionally activating a lateral teeth subsystem rhythm with 0.1 μM (6% of STGs) or 1 μM (43% of STGs), and reliably activating this rhythm using 5 μM (81% of STGs) (50). To evaluate the potential impact of hemolymph on this G-SIFamide action, we compared the gastric mill circuit response to incubating the isolated STG with 1 μM G-SIFamide in unfed or fed hemolymph versus 1 μM and 10 μM G-SIFamide in saline. Later, these conditions will be termed “1 μM-UH,” “1 μM-FH,” “1 μM-S,” and “10 μM-S,” respectively. As indicated earlier, we performed incubations instead of superfusions to accommodate the small hemolymph volumes obtained from each crab.

**Figure 4.**
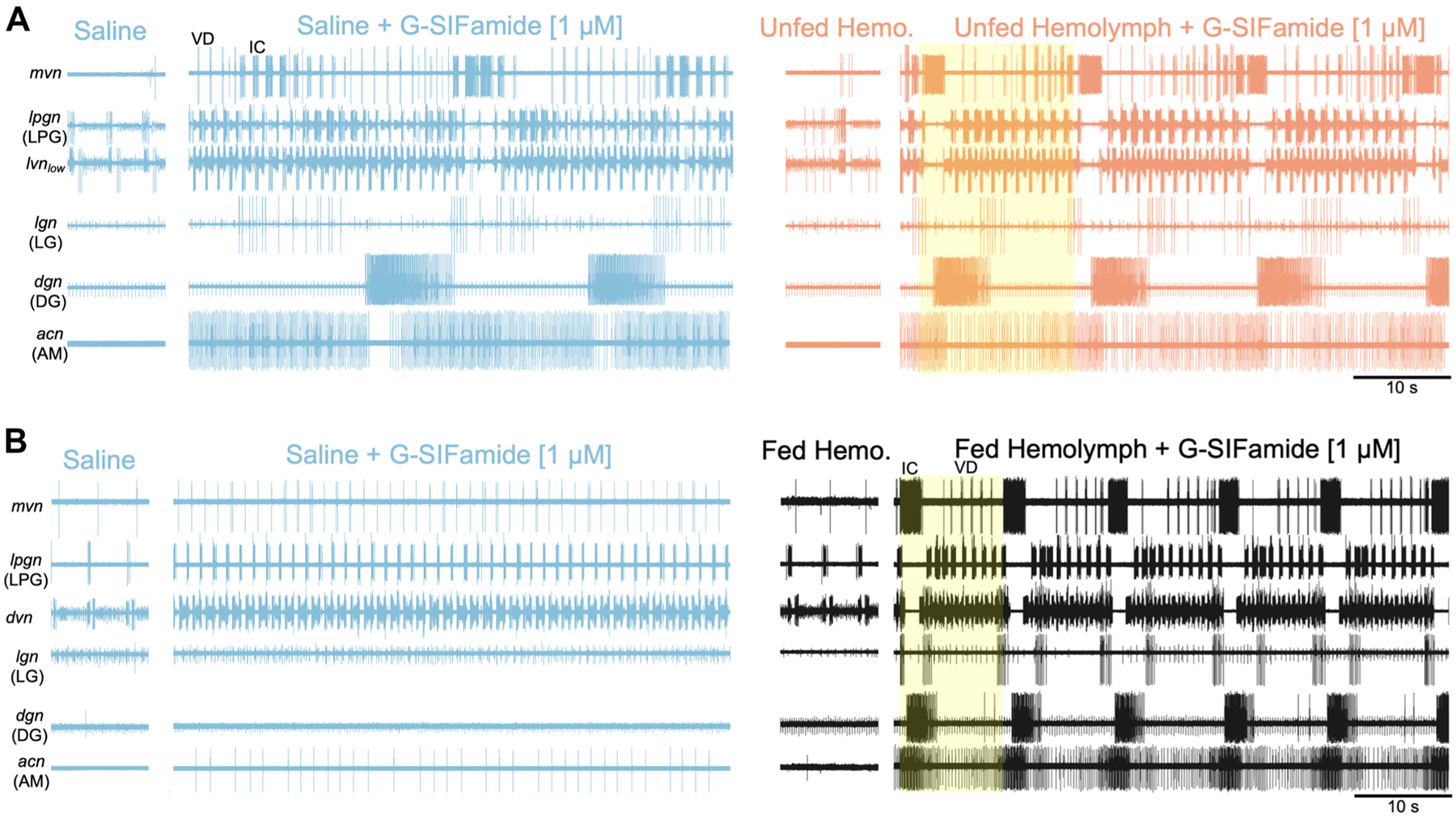
Example gastric mill rhythms elicited by 1 μM G-SIFamide in unfed and fed hemolymph. *A*: in the same preparation, 1 μM-unfed hemolymph (UH), but not 1 μM-saline (S), elicited the gastric mill rhythm. *Left*: relative to the preceding saline alone incubation, 1 μM-S elicited periodically strengthened inferior cardiac (IC) neuron activity which retained its pyloric rhythm-timed pattern and weakened but did not interrupt the pyloric rhythm. The pyloric rhythm was also more vigorous relative to the preceding saline incubation. *Right*: incubation with UH alone slowed the pyloric rhythm, whereas 1 μM-UH elicited a vigorous pyloric rhythm in addition to the gastric mill rhythm. The shaded region indicates one complete gastric mill rhythm cycle. *B*: in a different preparation, 1 μM-S increased the pyloric rhythm cycle frequency, whereas 1 μM-fed hemolymph (FH) elicited the gastric mill rhythm. Note that the gastric mill rhythm in 1 μM-FH was faster than in 1 μM-UH. Shaded region as in *A*.

Incubating the STG with G-SIFamide activated the gastric mill rhythm during all four of our experimental conditions (Figs. 2 and 4; Supplemental Fig. S1 and Supplemental Fig. S3, A and B). However, the occurrence of this rhythm was considerably more frequent during 1 μM-UH, 1 μM-FH, and 10 μM-S than during 1 μM-S (see Data Analysis). As described earlier, we defined each gastric mill rhythm cycle as spanning the onset of successive long duration (≥1 s) IC neuron bursts, as these bursts were the most consistently vigorous, rhythmically active component across these rhythms. The pyloric rhythm-timed IC bursts were considerably briefer (≤0.25 s). We further defined these rhythms as repeating cycles during which the neurons LG, AM, DG, and LPG generated rhythmic bursts coordinated with the long IC bursts, along with IC burst-associated interruptions of the pyloric rhythm.

**Figure 5.**
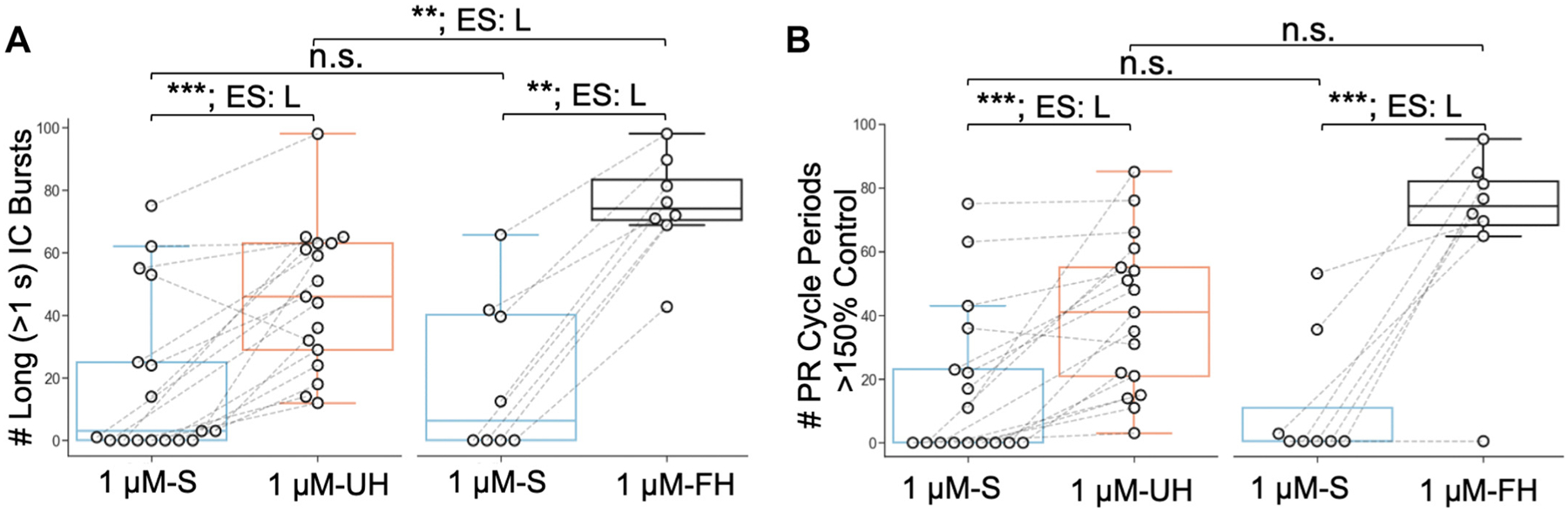
More long inferior cardiac (IC) bursts and associated pyloric rhythm interruptions occurred during 1 μM G-SIFamide incubations in unfed (UH) and fed hemolymph (FH) than in saline (S) during 900-s incubations. *A*: box and whisker plots show an increased mean number of long (>1 s) IC bursts per incubation in both 1 μM-UH (*left*) and 1 μM-FH (*right*) vs. 1 μM-S from the same preparation. There were also more long IC bursts in 1 μM-FH than 1 μM-UH. The associated effect sizes (ES) indicate large differences (1 μM-S vs. 1 μM-UH: *W* = 0.78, *n* = 17; 1 μM-S vs. 1 μM-FH: *W* = 1.0, *n* = 8, Kendall’s coefficient of concordance). Dashed lines connect data from the same preparation. N.S. (not significant), *P* > 0.05; **P < 0.01; ***P < 0.001, Friedman χ^2^; 1 μM-S vs. 1 μM-S: *P* = 0.9, and 1 μM-UH vs. 1 μM-FH: *P* = 0.003, RBC = 0.75; Mann–Whitney *U* test, rank biserial correlation. *B*: box and whisker plots show an increased mean number of pyloric rhythm interruptions per incubation in both 1 μM-UH vs. 1 μM-S (*W* = 0.78, *n* = 17) (*left*) and 1 μM-FH vs. 1 μM-S (*W* = 0.88, *n* = 8) (*right*). 1 μM-S vs. 1 μM-S: *P* = 0.6, and 1 μM-UH vs. 1 μM-FH: *P* = 0.08; Mann–Whitney *U* test. Dashed lines and statistics as in *A*.

The increased effectiveness of 1 μM G-SIFamide in activating the gastric mill rhythm when it was incubated in unfed or fed hemolymph is evident in the considerably increased number of prolonged IC bursts relative to the 1 μM-S incubations from the same preparations (1 μM-S: 18.5 ± 26.1; 1 μM-UH: 45.9 ± 23.0; *n* = 17, *P* < 0.0003, *W* = 0.78; 1 μM-S: 19.1 ± 24.6, 1 μM-FH: 71.9 ± 15.8, *n* = 8, *P* < 0.005, *W* = 1.0; Friedman χ^2^ test, Kendall’s coefficient of concordance) (Fig. 5A). This was also the case for the number of IC burst-associated delays of >150% relative to the control pyloric cycle period (1 μM-S: 17.1 ± 24.0, 1 μM-UH: 40.5 ± 24.2, *n* = 17, *P* < 0.0003, *W* = 0.78; 1 μM-S: 9.6 ± 17.7, 1 μM-FH: 57.8 ± 24.7, *n* = 8, *P* < 0.008, *W* = 0.88; Friedman χ^2^ test, Kendall’s coefficient of concordance) (Fig. 5B).

To determine whether the condition under which the peptide was applied had an impact on *1*) the frequency of occurrence of the gastric mill rhythm, and/or *2*) the parameters of the associated neuronal activity, we performed an in-depth characterization of the gastric mill circuit response to each condition. Specifically, we extracted a set of features (e.g., inter-spike interval, firing rate, and phase relative to gastric mill rhythm-timed IC bursts; see materials and methods) from most gastric mill neurons, and the pyloric neuron PD, across four successive and nonoverlapping 200-s segments per incubation, starting 60 s after incubation onset. Thus, this analysis covered 800/840 s (95%) of each 900 s incubation, starting 60 s after incubation onset. For context, there were ~10–15 gastric mill cycles during any one segment when there was a persistent gastric mill rhythm, insofar as the gastric mill cycle period was ~13–20 s (Table 1). We then performed a principal component analysis (components = 10) on our set of features, and used *k*-means (*n* = 3) to cluster the resulting data into one of three clusters based upon minimizing the distance between data points in a cluster with their centroid. Thus, unless otherwise indicated, the data analyses presented in the following sections were performed at the level of the segments, with “*n*” representing the number of segments (e.g., Figs. 7, 8, 9, 10, and 11, Tables 1 and 2).

**Figure 6.**
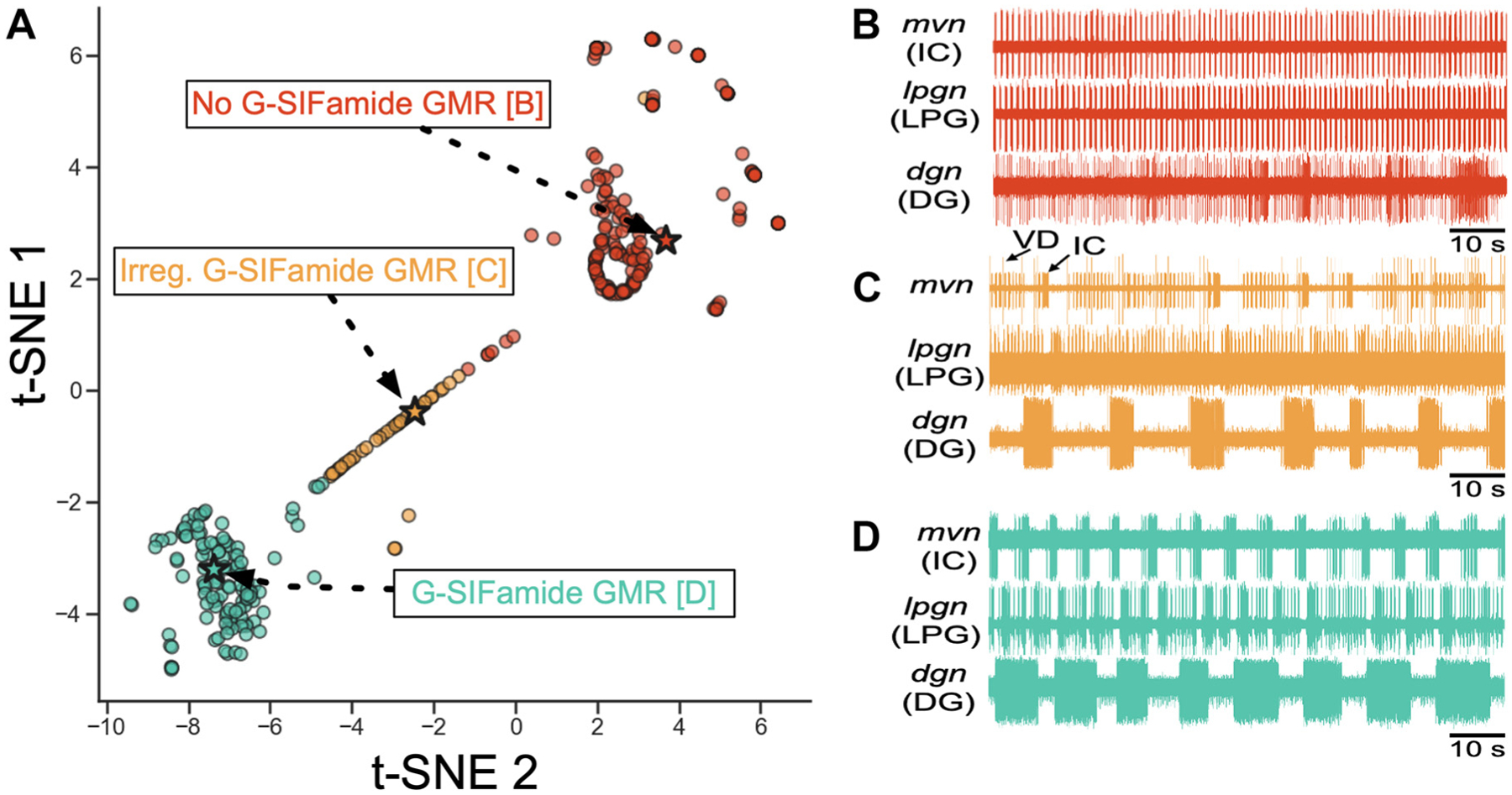
Relationship of the gastric mill circuit response to 1 μM G-SIFamide across incubations and conditions. *A*: across conditions [1 μM-saline (S), 10 μM-S, 1 μM-unfed hemolymph (UH), 1 μM-fed hemolymph (FH)], the gastric mill circuit response to G-SIFamide was clustered into three groups, including “no gastric mill rhythm” (no GMR; i.e. gastric mill neurons exhibited no coordinated activity), an “irregular gastric mill rhythm” (the gastric mill rhythm was only intermittently active, cycled slowly [cycle period > 40 s] and/or did not include coordinated activity in all neurons), and a “gastric mill rhythm” (gastric mill rhythm was continually active at cycle periods of ~20 s). *B*–*D*: example responses from each cluster, indicated by arrows and starred markers in *A*. Note that inferior cardiac (IC) neuron activity was exclusively pyloric rhythm-timed in *B*, exhibited intermittent prolonged bursts in *C*, and exhibited regular repeating long bursts in *D*. *D* also displays an example of a gastric mill rhythm wherein there was one DG burst for every two gastric mill cycles. DG, dorsal gastric; *dgn*, dorsal gastric n.; *lpgn*, lateral posterior gastric n.; *mvn*, medial ventricular n.

Our *k*-means clustering revealed three clusters that exhibited only minor differences within a cluster but major differences between clusters (Fig. 6). The resulting clusters effectively captured whether each included segment displayed *1*) a gastric mill rhythm, *2*) an irregular gastric mill rhythm, or *3*) no gastric mill rhythm. Gastric mill rhythm segments exhibited one of several distinct phase relationships between the IC and DG neurons. For example, although there was often a consistent 1:1 relationship of long IC to DG bursts across gastric mill cycles (Figs. 2 and 4; Supplemental Fig. S1), in some incubations the DG burst consistently occurred during every other gastric mill cycle (Fig. 6D; Supplemental Fig. S2). Irregular rhythms most commonly exhibited slow [cycle period >40 s] and inconsistent long IC bursting (Cycle Period coefficient of variation—gastric mill rhythm vs. irregular gastric mill rhythm: 1 μM-UH, 0.18 vs. 0.37, *n* = 11 vs. 23; 1 μM-FH, 0.13 vs. 0.31, *n* = 5 vs. 38; 10 μM-S, 0.26 vs. 0.44, *n* = 9 vs. 42). Less frequently, the irregular rhythms exhibited absent or erratic activity in the DG, LG, or AM neuron.

### Hemolymph Increases the Occurrence of the Gastric Mill Rhythm

**Figure 7.**
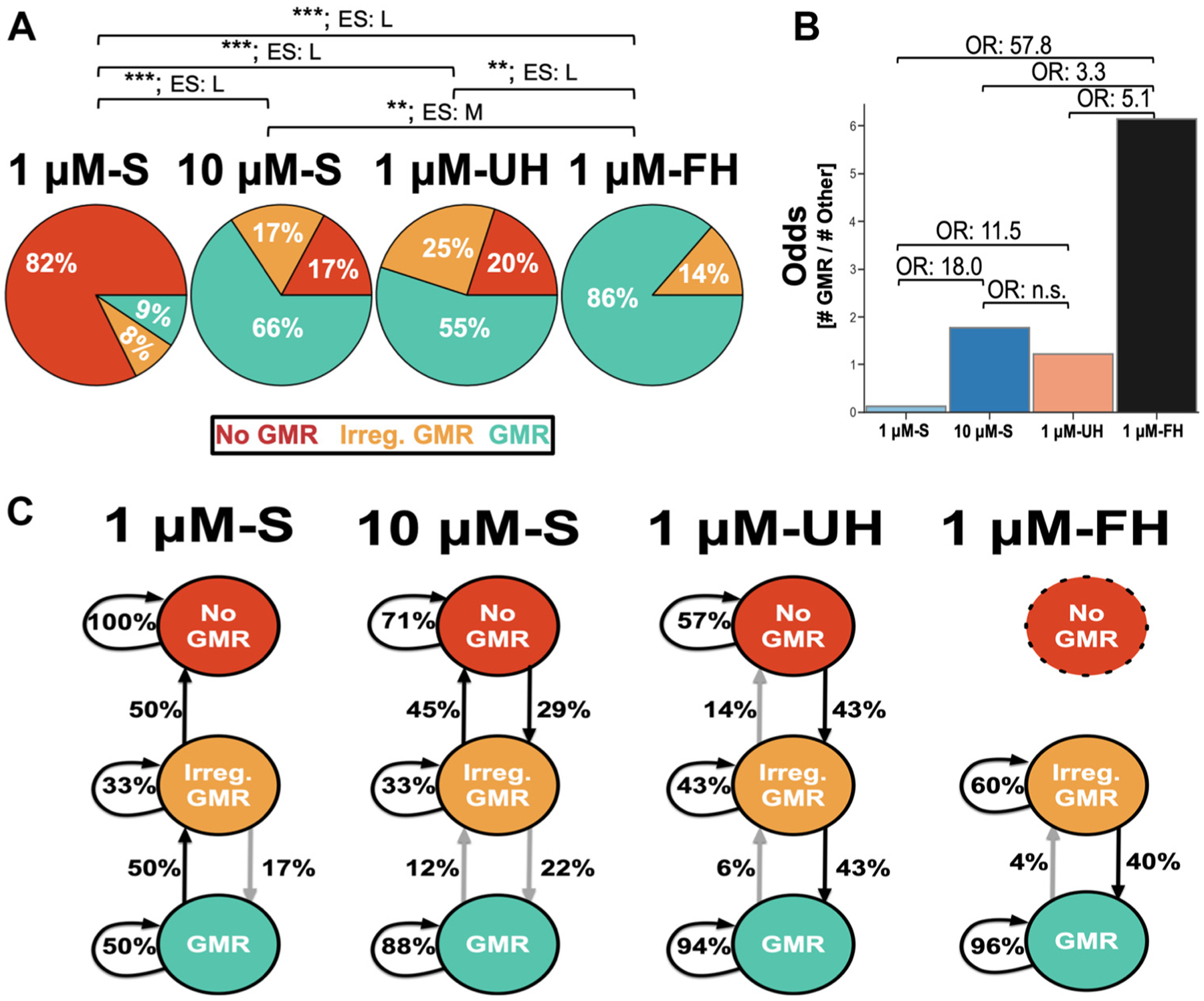
Comparison of the gastric mill circuit response to G-SIFamide incubations in four experimental conditions. *A*: the distribution of outcomes (no gastric mill rhythm, irregular gastric mill rhythm, gastric mill rhythm) per 200-s segment favored no gastric mill rhythm in 1 μM-saline (S), whereas it favored a gastric mill rhythm in the other three conditions. The outcome distribution in 1 μM-S differed from the other three conditions (***P < 0.001, χ^2^ test), with each comparison exhibiting a large effect size (ES range: 0.6–0.8, Cramér’s V). The outcome distribution for 1 μM-fed hemolymph (FH) is also distinct from the other three conditions (**P < 0.01, χ^2^ test; ES range: 0.3–0.8, Cramér’s V), while the distribution is comparable between 1 μM-unfed hemolymph (UH) and 10 μM-S (*P* > 0.05, χ^2^ test). *B*: The odds ratio between the outcome per segment being a gastric mill rhythm (GMR) vs. the other two possible outcomes (i.e., an irregular rhythm, no rhythm) is distinct, and large (~10- to ~50-fold), between 1 μM-S and the other three conditions. The odds ratio is also three- to fivefold higher for 1 μM-FH relative to 1 μM-UH and 10 μM-S, whereas the ratio between the latter two conditions was comparable. *C*: outcome transitions between 200 s segments for the three experimental conditions. For example, during 1 μM-S every segment during which there was no gastric mill rhythm was followed by the next segment exhibiting the same outcome. In contrast, in the 1 μM-UH and 10 μM-S conditions some subsequent segments transitioned from no gastric mill rhythm to an irregular gastric mill rhythm (1 μM-UH: 43%; 10 μM-S: 29%).

As shown in Fig. 7A, the distribution of outcomes per segment in 1 μM-S was different from that of the other three conditions (*P* < 0.001, χ^2^ test), and the associated effect size (0.6–0.8, Cramér’s V) indicates a large magnitude difference in the proportions for each comparison. For example, incubations with 1 μM-S primarily resulted in segments (82% of segments) displaying no gastric mill rhythm while, in contrast, incubations with 1 μM-FH displayed a gastric mill rhythm in 86% of segments. The distribution for 1 μM-FH was also different from that of the other three conditions, including the absence of “no gastric mill rhythms,” and displayed a middle to large effect size value (0.3–0.8, Cramér’s V; Fig. 7A). In contrast, there was no difference in the distribution of outcomes between the 1 μM-UH and 10 μM-S conditions (Fig. 7A). These outcomes and those presented below indicate that both hemolymph conditions enable the gastric mill circuit to respond to 1 μM G-SIFamide as if the peptide was being applied at a higher concentration (~10 μM G-SIFamide), with fed hemolymph being more effective than unfed hemolymph.

Figure 7A also shows that a higher percentage of segments exhibited a gastric mill rhythm when the STG was incubated with 1 μM-UH, 1 μM-FH, or 10 μM-S rather than 1 μM-S. For example, 1 μM-S incubations elicited a gastric mill rhythm in only 9% of segments (9/96 segments from 4/24 [17%] STGs), with an irregular rhythm occurring in another 8% of segments and no gastric mill rhythm in the remaining 82% of segments. In comparison, a gastric mill rhythm occurred in 55% of 1 μM-UH segments (21/40 segments from 7/10 [70%] STGs), 86% of 1 μM-FH segments (38/44 segments from 11/11 [100%] STGs), and 66% of 10 μM-S segments (42/64 segments from 12/16 [75%] STGs) (Fig. 7A). There were also irregular rhythms in the latter three conditions (1 μM-UH: 25%; 1 μM-FH: 14%; 10 μM-S: 17%) and no gastric mill rhythm in some segments during incubations with 1 μM-UH (20%) and 10 μM-S (17%). All segments during 1 μM-FH activated either a gastric mill rhythm or irregular gastric mill rhythm (Fig. 7A).

We calculated the odds of a gastric mill rhythm occurring (i.e., no. of segments with gastric mill rhythms ÷ no. of segments with irregular or no gastric mill rhythm) as a first step to testing for differences in gastric mill rhythm occurrence across conditions. We then constructed an odds ratio between groups (e.g., 1 μM-FH Odds ÷ 1 μM-UH Odds) with a 95% confidence interval (CI; corresponding to α = 0.05) (Fig. 7B). The CI falling entirely above or below a value of 1.0 was deemed significant, corresponding to *P* ≤ 0.05 (75). The distribution of outcomes across our conditions results in there being, relative to 1 μM-S, higher odds that a gastric mill rhythm occurs in a 200-s segment during 1 μM-UH (11.5-fold increase: 95% CI: 4.3–33.6), 1 μM-FH (57.8-fold increase: 95% CI: 18.4–217.4), and 10 μM-S (18.0-fold increase: 95% CI: 7.3–48.8) (Fig. 7B). The odds of this occurrence in 1 μM-FH were also higher than in 1 μM-UH (5.1-fold increase: 95% CI: 1.6–18.1) and 10 μM-S (3.3-fold increase: 95% CI: 1.1–11.0). In contrast, there were comparable odds of the gastric mill rhythm occurring in 1 μM-UH or 10 μM-S (95% CI: 0.3–1.6).

In STGs where at least one segment displayed a gastric mill rhythm, that rhythm persisted across segments more often in both hemolymph conditions (1 μM-UH: 94%; 1 μM-FH: 96%) and with 10 μM-S (88%) than with 1 μM-S (50%) (Fig. 7C). Furthermore, in the subset of experiments where a gastric mill rhythm occurred in at least one segment, this rhythm persisted for the entire incubation (i.e., all four segments) in only 1 of 4 STGs (25%) during 1 μM-S. In comparison, the rhythm persisted for the entire incubation more frequently in 1 μM-UH (4/7 STGs [57%]), 1 μM-FH (8/11 STGs [73%]), and 10 μM-S (8/12 STGs [67%]). When a segment instead displayed no gastric mill rhythm, it was always followed by another segment with no gastric mill rhythm during 1 μM-S incubations (*n* = 58/58, 100%), whereas there were transitions across segments from no rhythm to an irregular rhythm in subsets of experiments with 1 μM-UH (*n* = 3/7, 43%) and 10 μM-S (*n* = 2/9, 29%) (Fig. 7C). Interestingly, in all four conditions transitions only occurred between adjacent clusters (i.e., gastric mill rhythm into/from irregular rhythm, and no gastric mill rhythm into/from irregular rhythm) (Fig. 7C).

We also determined whether there was a lasting influence of the first 1 μM-S incubation by following it in some experiments with a second 1 μM-S incubation. In these experiments, the distribution of gastric mill circuit responses (i.e., gastric mill rhythm, irregular gastric mill rhythm, no gastric mill rhythm) that occurred during each incubation was comparable (*P* = 0.42, *n* = 40 segments [10 STGs], χ^2^ test).

### Phase Relationships during the Gastric Mill Rhythm

**Figure 8.**
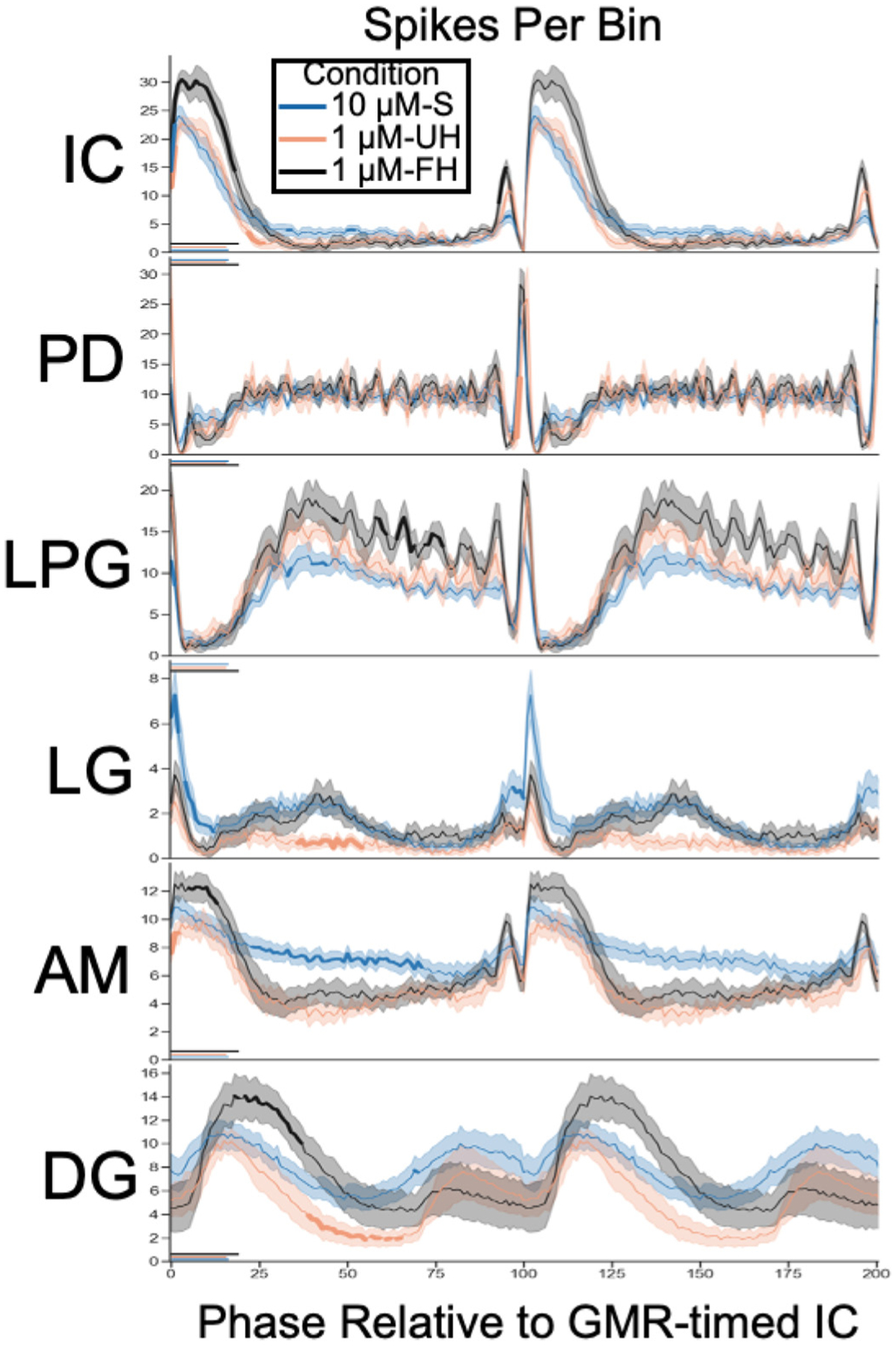
Phase relationships during G-SIFamide-elicited gastric mill rhythms in saline and hemolymph. Plotted is the average number of spikes per normalized bin duration, for each neuron shown, across the gastric mill rhythm (GMR) cycle during incubations with 1 μM-unfed hemolymph (UH), 1 μM-fed hemolymph (FH), and 10 μM-saline (S). Each cycle, which extends from the start of consecutive long inferior cardiac (IC) neuron bursts, was divided into 100 equal duration bins. The three colored horizontal lines associated with each plot represent the mean IC neuron duty cycle under each condition (10 μM-S: 15.1 ± 1.1%; 1 μM-UH: 15.2 ± 1.4%; 1 μM-FH: 19.0 ± 1.8%; 1 μM-FH vs. 1 μM-UH, *P* < 0.001; 1 μM-FH vs. 10 μM-S, *P* < 0.001, 1 μM-UH vs. 10 μM-S, *P* > 0.05; confidence intervals on differences between group means). Thicker lines within each plot, during the first of the two cycles displayed, indicate regions where the labeled bins are different (*P* < 0.01, confidence intervals on differences between group means) from the other two conditions across at least two consecutive bins. Faded colors tracking each line indicate the SE, calculated on the mean value across all segments within each preparation. AM, anterior median; DG, dorsal gastric; LG, lateral gastric; LPG, lateral posterior gastric; PD, pyloric dilator.

We also determined whether the resulting gastric mill rhythm pattern was comparable during our three experimental conditions (1 μM-UH, 1 μM-FH, 10 μM-S). As shown earlier, the gastric mill rhythm during the G-SIFamide incubations included activity in at least four gastric mill neurons coordinated to the long IC burst-timed activity, plus the long IC burst-timed interruption of the pyloric rhythm (Figs. 2 and 4; Supplemental Fig. S1). Two additional STG neurons, GM and MG, did not participate in these rhythms despite doing so in some, but not all, other versions of the gastric mill rhythm (49, 81, 86, 91). Across preparations and conditions, the IC neuron burst extended for the first ~20% of the gastric mill cycle, during which there was an overlapping but brief LG neuron peak in the number of spikes/bin at cycle onset, an AM neuron peak that tracked the IC burst, and the pyloric rhythm interruption, which is represented here by the drop in LPG and PD neuron activity (Fig. 8). The prolonged IC burst was often preceded by a briefer burst that contained more IC spikes than during the preceding pyloric cycles. This “prepeak” was also evident in the AM and, to a lesser extent, LG neuron. DG neuron activity peaked near the end of the IC burst and persisted until the cycle mid-point (Fig. 8). A second DG peak is evident from ~75%–100% of the cycle, but it does not represent a second DG burst during each gastric mill cycle. Instead, this second peak represents the presence of a shifting IC:DG phase relationship during some gastric mill rhythms, as well as a consistent but distinct phase relationship during some incubations (e.g., Fig. 6D; Supplemental Fig. S2).

The PD and LPG neurons exhibited a sharp, large peak preceding each gastric mill cycle transition, indicative of each long IC burst consistently occurring after an episode of the inhibition it receives from the pyloric pacemaker neurons (note the corresponding drop in IC activity) (Fig. 8). Following that inhibition, there was a drop in PD and LPG activity associated with the long IC burst. PD then resumed its steady pyloric rhythm-related activity while the pyloric rhythm-timed LPG activity climbed, peaking as the DG burst was waning at the ~35%–50% point of the cycle (Fig. 8). During some gastric mill cycles in most preparations, this increased LPG activity included its burst spanning more than one pyloric cycle (Fig. 6, C and D; Supplemental Fig. S3). In addition, while the PD neuron duty cycle remained at a constant level of ~20% across the pyloric rhythm cycles between the long IC bursts, the coactive LPG duty cycle expanded considerably (>twofold the PD duty cycle; Supplemental Fig. S3).

The phase relationship among these neurons was broadly similar across incubation conditions but included some condition-specific distinctions (Fig. 8). For example, during 1 μM-FH the peaks in IC, AM and DG (first peak) were higher than in the other two conditions, and the IC duty cycle was increased (1 μM-FH vs. 1 μM-UH: *P* < 0.001; 1 μM-FH vs. 10 μM-S: *P* < 0.001; 1 μM-UH vs. 10 μM-S: *P* = ns; CI from difference of means) (Table 1). A 1:1 burst relationship between IC and DG also occurred more frequently in 1 μM-FH than the other two conditions (Supplemental Fig. S2).

During 1 μM-UH, the postpeak level for DG was lower and there was no evident second peak in LG, whereas during 10 μM-S the postpeak level in AM was higher, giving AM a more tonic-appearing firing pattern than in either hemolymph condition (Fig. 2B, Fig. 4, and Fig. 8). The IC neuron “prepeak” was also less pronounced in 10 μM-S than in the other two conditions during the two bins that contain the prepeak spikes (*P* < 0.01; CI from difference of group means). Overall, the gastric mill rhythm pattern occurring during 1 μM-UH and 1 μM-FH was similar to that during 10 μM-S, with the few differences in peak amplitudes among some neurons occurring in one or another of the three conditions.

### Condition-Specific Neuronal Activity during the Gastric Mill Rhythm

**Figure 9.**
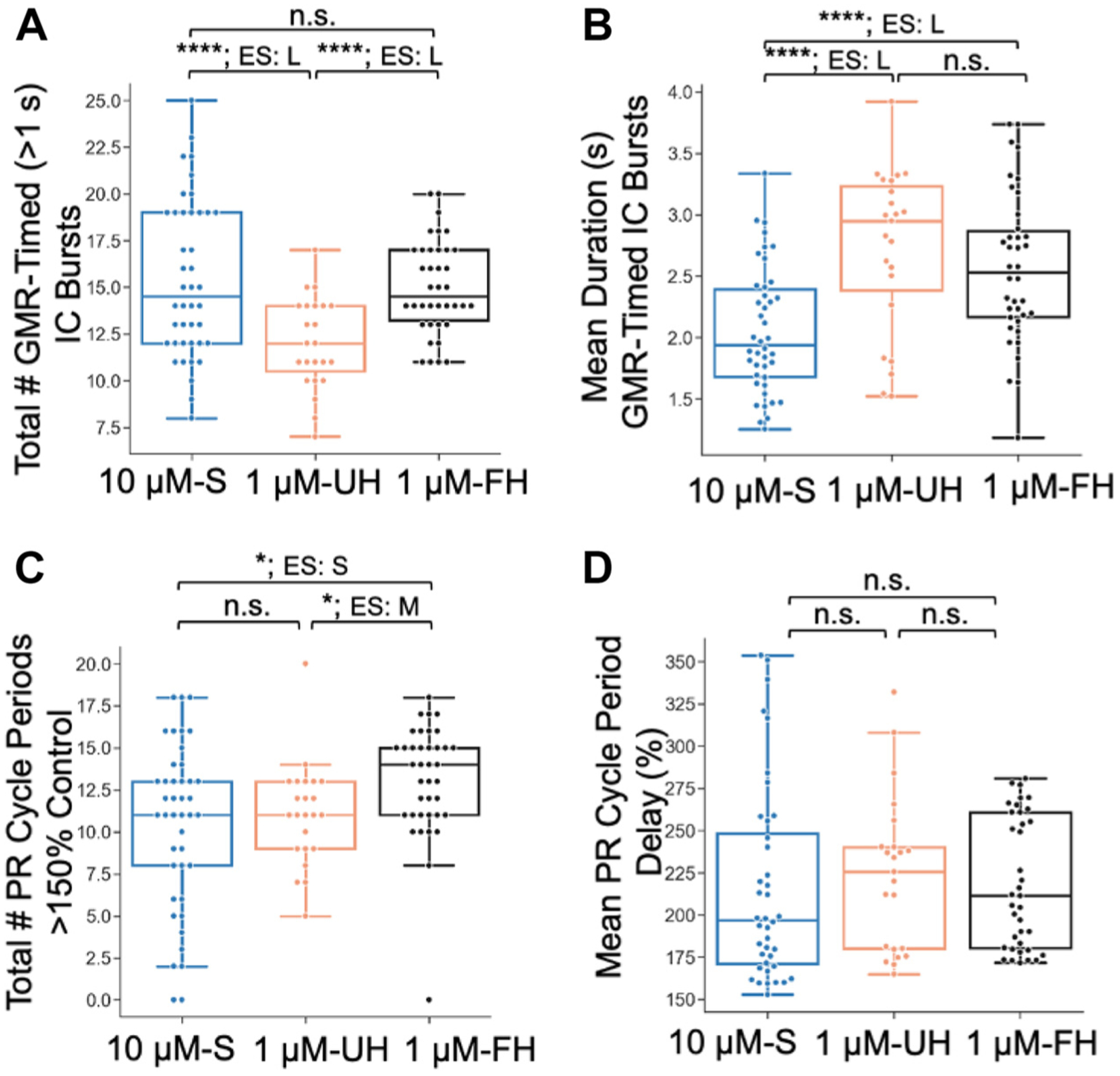
Comparison of inferior cardiac (IC) burst-related parameters during G-SIFamide gastric mill rhythm segments across conditions. *A*–*D*: box and whisker plots comparing, across conditions [10 μM-saline (S), *n* = 42 segments, 12 stomatogastric ganglia (STGs); 1 μM-unfed hemolymph (UH), *n* = 25 segments, 8 STGs; 1 μM-fed hemolymph (FH), *n* = 38 segments, 11 STGs], the total number of long IC bursts (*A*); mean duration of the long IC bursts (*B*); total number of IC burst-associated interruptions in the pyloric rhythm (PR) (*C*), and; mean duration (% relative to control pyloric cycle period) (*D*) of the interrupted pyloric rhythm. See Fig. 5 for definitions of each aspect of the box and whisker. Statistics: n.s. *P* > 0.05; *P < 0.05; ****P < 0.0001, confidence intervals on differences between group means; *d* determined using Cohen’s *d* (see Table 1 for Cohen’s *d* values).

We also assessed whether the two hemolymph conditions differently altered any activity parameters during the G-SIFamide gastric mill rhythm, and how their outcomes compared with those in 10 μM-S. There were, indeed, notable differences between 1 μM-FH and 1 μM-UH in several activity parameters, including most IC neuron-related parameters (Fig. 9; Tables 1 and 2). For example, in 1 μM-FH there was a larger IC duty cycle (see aforementioned), a briefer cycle period (*P* < 0.00001, Cohen’s *d* = −1.5), and a higher IC firing rate (*P* < 0.00001, Cohen’s *d* = 1.3). There were also a larger number of gastric mill rhythm-timed IC Bursts (*P* < 0.00001, Cohen’s *d* = 0.9), and an increased number of interrupted pyloric rhythm cycles (*P* < 0.01, Cohen’s *d* = 0.4) (Fig. 9, A and C, Table 1).

There were fewer differences between the two hemolymph conditions in the activity parameters of other gastric mill neurons. Specifically, in 1 μM-FH there were more LG neuron spikes per burst (*P* < 0.001, Cohen’s *d* = 1.0), and a higher LG firing rate (*P* = 0.00001, Cohen’s *d* = 1.4) (Table 2). The average baseline pyloric rhythm (determined from pyloric rhythm cycles exhibiting <10 IC spikes; see materials and methods) was also slower in 1 μM-UH than 1 μM-FH (*P* < 0.00001, Cohen’s *d* = −1.4), and exhibited longer pyloric rhythm interruptions (*P* < 0.001, Cohen’s *d* = −1.1) (Tables 1 and 2).

There were also some distinctions in gastric mill neuron burst parameters during gastric mill rhythms elicited by 1 μM-FH versus 10 μM-S. For example, in 1 μM-FH the IC neuron exhibited longer duration bursts (*P* < 0.00001, Cohen’s *d* = 0.8), more spikes per burst (*P* < 0.0001, Cohen’s *d* = 1.0), a higher firing rate (*P* < 0.00001, Cohen’s *d* = 1.2), and larger duty cycle (*P* < 0.001, Cohen’s *d* = 0.6) (Fig. 9; Table 1). The 1 μM-FH incubations also exhibited more IC-associated pyloric rhythm interruptions (*P* < 0.01, Cohen’s *d* = 0.4) (Fig. 9; Table 1).

In contrast, compared with 1 μM-FH, AM produced more spikes per burst in 10 μM-S incubations (*P* < 0.00001, Cohen’s *d* = −1.0). DG also produced longer bursts (Table 2). There were no additional difference between these two conditions with respect to other analyzed gastric mill or pyloric rhythm parameters (Tables 1 and 2).

Compared with 1 μM-UH, incubations in 10 μM-S produced briefer IC bursts (*P* < 0.00001, Cohen’s *d* = 1.2), fewer IC spikes per burst (*P* < 0.0001, Cohen’s *d* = 1.0), and briefer IC-associated pyloric rhythm interruptions (*P* < 0.0001, Cohen’s *d* = 1.2) (Fig. 9; Table 1).

Some parameters were instead enhanced to a greater extent in 10 μM-S relative to 1 μM-UH. These included more long IC bursts (*P* < 0.0001, Cohen’s *d* = −0.9), and a faster gastric mill rhythm (*P* < 0.001, Cohen’s *d* = 1.5) (Fig. 9; Table 1). There were also more LG neuron spikes per burst, with a higher intraburst firing rate and a longer burst duration (Table 2). Finally, the AM neuron firing rate was higher and the baseline pyloric rhythm was faster in 10 μM-S than 1 μM-UH (Table 2). Nine additional burst parameters were not different between 10 μM-S and 1 μM-UH (Table 2). Collectively, there were no parameters for which there was a significant difference across all three comparisons, and only 3 of 19 (16%) parameters where comparisons failed to reveal any differences (Tables 1 and 2). Thus, overall, there were similarities and differences between the two hemolymph conditions and between each of them and the 10 μM-S condition. These outcomes add to those in the previous section supporting the hypothesis that hemolymph enhances the G-SIFamide action on the gastric mill circuit similarly, albeit not identically, to a 10-fold increase in the peptide concentration.

Previous work (50) established a slowing of the pyloric rhythm during long IC bursts elicited by superfusing 5 μM G-SIFamide in saline. Here, we showed that a distinct association occurred between long IC bursts and pyloric rhythm slowing across our three experimental conditions. Specifically, there were more long IC bursts during incubations with 10 μM-S and 1 μM-FH than with 1 μM-UH, but 10 μM-S elicited fewer PR cycle periods >150% of their control value than 1 μM-FH and a comparable number to 1 μM-UH (Fig. 9; Table 1). To better understand these results, we performed additional analyses.

**Figure 10.**
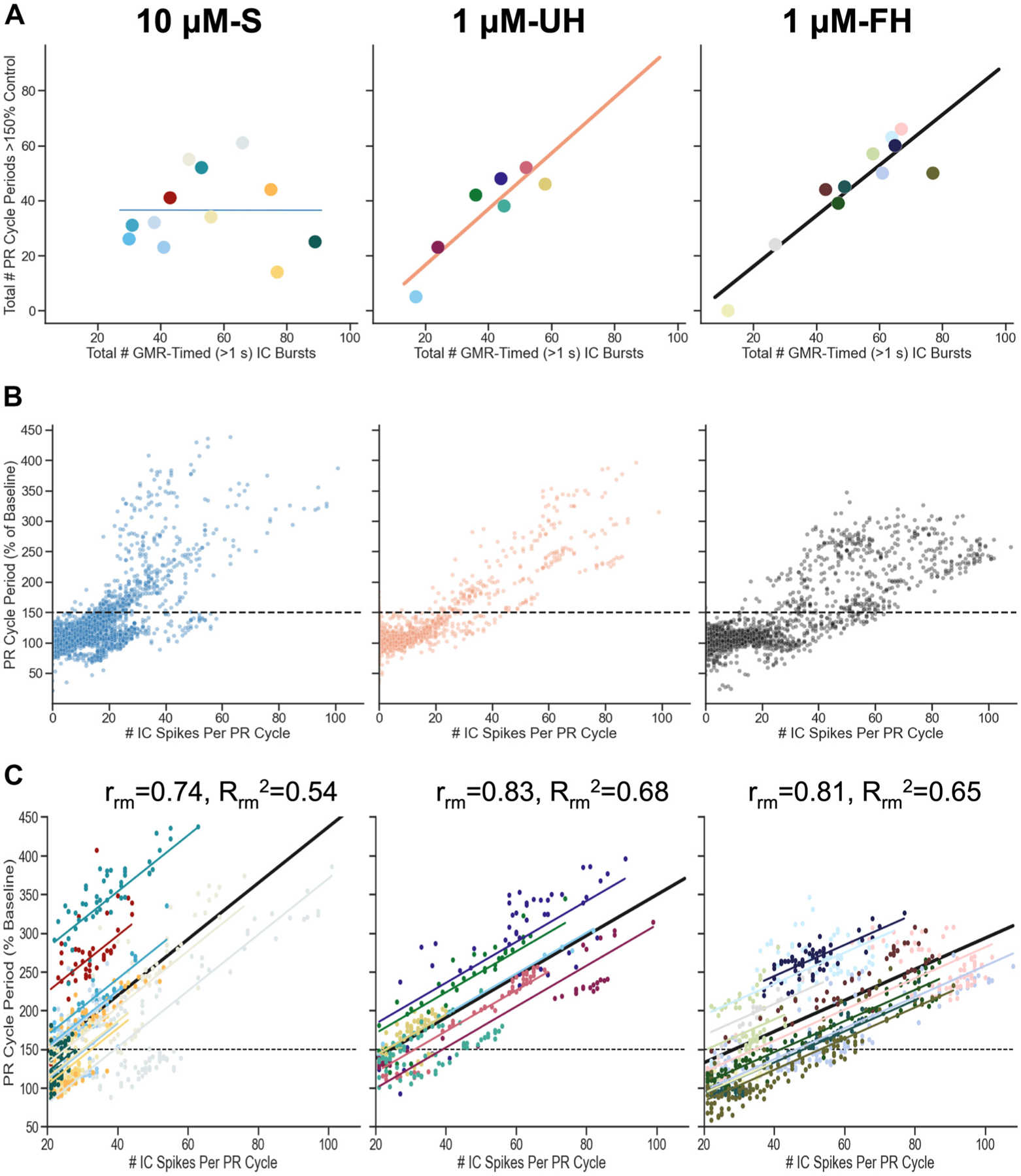
Correlation of inferior cardiac (IC) activity and pyloric rhythm delays during the G-SIFamide gastric mill rhythm across conditions. *A*: number (#) of pyloric rhythm cycle periods >150% control value as a function of no. gastric mill rhythm-timed IC bursts, aggregated across all gastric mill rhythm segments in each preparation (colored circles). Linear regression analysis indicates a positive correlation of these parameters in the 1 μM-fed hemolymph (FH) (*P* = 0.00009, *n* = 11) and 1 μM-unfed hemolymph (UH) (*P* = 0.001, *n* = 7), but not 10 μM-saline (S) (*P* > 0.05, *n* = 12), condition. *B*: scatterplot of the pyloric rhythm cycle period (% of baseline cycle period) as a function of the number of IC spikes within that cycle [10 μM-S: 7,502 pyloric rhythm (PR) cycles, 12 stomatogastric ganglia (STGs); 1 μM-UH: 3,033 PR cycles, 7 STGs; 1 μM-FH: 6,231 PR cycles, 11 STGs]. Dashed line, 150% of baseline pyloric cycle period. *C*: repeated measures correlation for the data from *B*, sorted by STG (colors correspond to those in *A*) and overall group lines (dark black) in each condition for IC bursts with ≥20 spikes (10 μM-S: 12 STGs; 1 μM-UH: 7 STGs; 1 μM-FH: 11 STGs). All groups significantly correlated; *P* < 0.00001. Dashed line, 150% of baseline pyloric cycle period.

First, we determined whether each condition had a similar relationship between long IC bursts and pyloric rhythm slowing. Specifically, we performed linear regressions predicting the number of pyloric rhythm cycles slowed >150% of the baseline cycle period based on the number of long IC bursts. The number of these bursts were not predictive of the pyloric rhythm response for 10 μM-S (*P* = 0.99, *n* = 12), but they were predictive for 1 μM-UH (*r* = 0.92, *R*^2^ = 0.85, *P* = 0.001, *n* = 7, linear regression), and 1 μM-FH (*r* = 0.91, *R*^2^ = 0.83, *P* = 0.00009, *n* = 11, linear regression) (Fig. 10A).

To determine whether the different outcome in Fig. 10A for the 10 μM-S condition relative to the two hemolymph conditions resulted from a distinct relationship between pyloric cycle period and IC neuron activity, we first plotted the normalized pyloric cycle period as a function of the number of IC spikes for all pyloric cycles during each incubation (10 μM-S: 7,502 PR cycles, 12 STGs; 1 μM-UH: 3,033 PR cycles, 7 STGs; 1 μM-FH: 6,231 PR cycles, 11 STGs) (Fig. 10B). As shown in Fig. 10B, the overall trajectory for this relationship was similar for all three conditions. Specifically, in all three conditions there were very few pyloric cycles that were >150% of the baseline cycle period when there were <20 IC spikes per cycle, whereas there were many such cycles when there were >20 IC spikes. Note that, for all three conditions, the average intraburst firing rate for the IC neuron ranged from 21 Hz (1 μM-UH, 10 μM-S) to 28 Hz (1 μM-FH) (Table 1). Therefore, the 20 IC spikes per cycle point on the *x*-axis of Fig. 10B approximates our defined minimum value for a “long IC burst” (i.e., >1 s duration).

It remained possible that Fig. 10B obscured a distinction in the pyloric cycle period change as a function of IC spiking in different STGs. Therefore, we characterized the overall within-individual association between the number of IC spikes and pyloric rhythm cycle period for each condition (10 μM-S, 1 μM-UH,1 μM-FH). For all three conditions, there was a positive relationship to this association, and all of these effects were considered large (10 μM-S: *r*_rm_ = 0.74, 95% CI = 0.7: 0.77, *P* = 7.9 × 10^−118^, *n* = 12; 1 μM-UH: *r*_rm_ = 0.83, 95% CI = 0.79: 0.86, *P* = 7.8 × 10^−77^, *n* = 7; 1 μM-FH: *r*_rm_ = 0.81, *P* = 1.6 × 10^−179^, 95% CI = 0.78: 0.83, *n* = 11; repeated measures correlation) (Fig. 10C). Consequently, whereas only the two hemolymph plus G-SIFamide incubations exhibited a relationship between the long IC bursts and pyloric rhythm cycles delayed >150% of the baseline cycle period (Fig. 10A), all three conditions displayed a consistently positive relationship between pyloric cycle period and IC neuron activity within individual STGs (Fig. 10C). These results suggest that the arbitrary values selected for IC burst duration (>1 s) and pyloric rhythm slowing (>150% of baseline) to evaluate the relationship between these two parameters across preparations is appropriate for the hemolymph conditions but not for the 10 μM-S condition.

### Condition-Specific Desensitizing Gastric Mill Circuit Response to G-SIFamide Incubation

**Figure 11.**
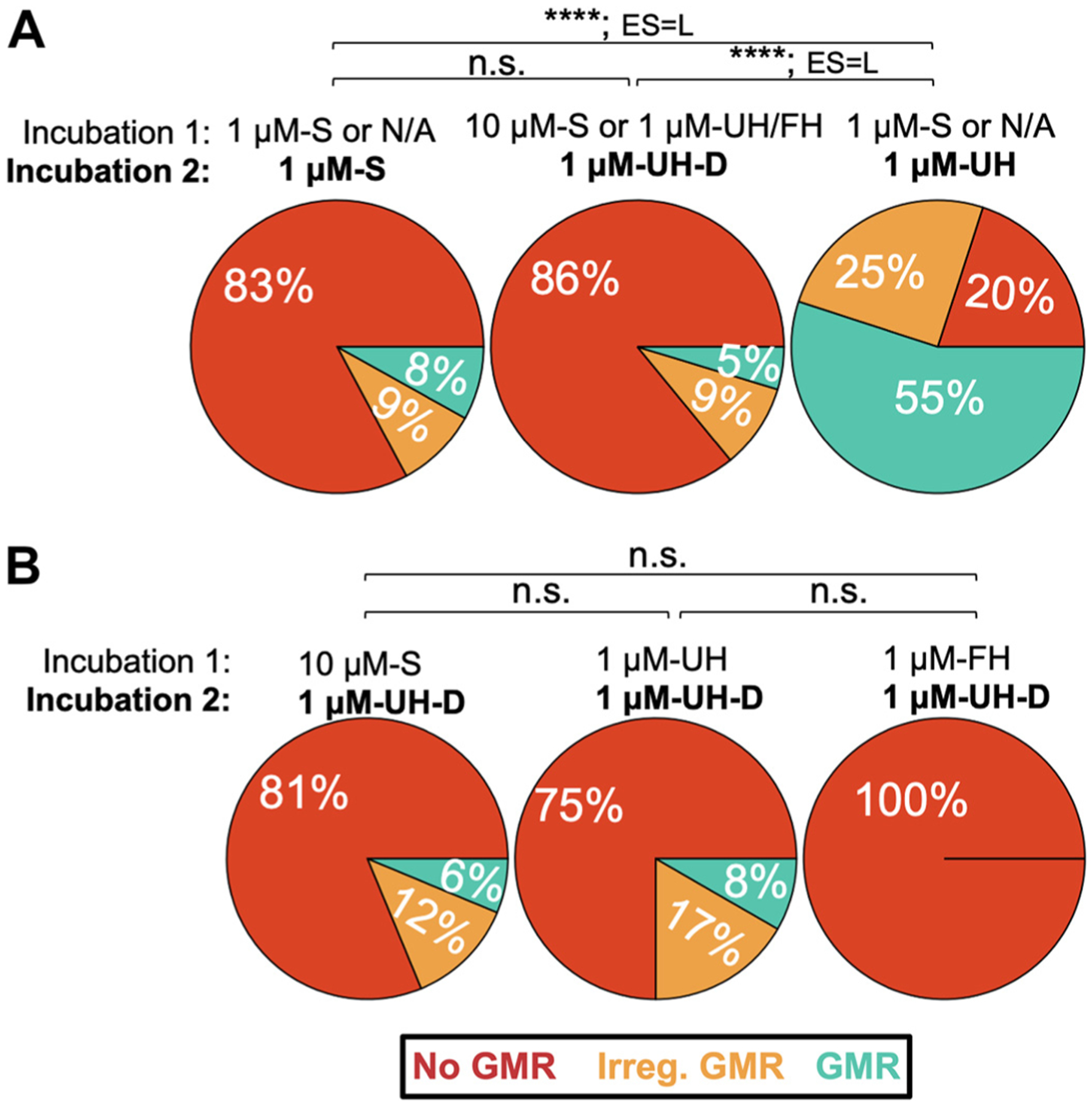
Desensitized gastric mill circuit response to 1 μM-unfed hemolymph (UH) following incubation with 1 μM G-SIFamide in either hemolymph or 10 μM-saline (S). *A*: the distribution of gastric mill circuit responses to 1 μM-UH-D (D = desensitized) following an initial incubation with either 10 μM-S, 1 μM-UH, or 1 μM-fed hemolymph (FH) was comparable to the response distribution in 1 μM-S, regardless of whether the 1 μM-S was Incubation-1 or Incubation-2 (following a first incubation in 1 μM-S). In contrast, the response distribution of 1 μM-UH-D was distinct from that of 1 μM-UH when it was used as either Incubation-1 or Incubation-2 (following a first incubation in 1 μM-S). The distribution of outcomes for 1 μM-UH-D represents the mean values from the three separate distributions shown in *B*. n.s. *P* > 0.05; ****P < 0.00001; χ^2^ test. N/A, Incubation-2 was not preceded by an Incubation-1. *B*: after an initial incubation (Incubation-1) with either 10 μM-S [*n* = 32 segments, 8 stomatogastric ganglia (STGs)], 1 μM-UH (*n* = 12 segments, 3 STGs) or 1 μM-FH (*n* = 20 segments, 5 STGs) and subsequent 1-h saline wash, segments (200 s) of incubation with 1 μM-UH resulted most often (Incubation-1: 10 μM-S, 1 μM-UH) or always (Incubation-1: 1 μM-FH) in no gastric mill rhythm, though there was no difference in the outcome across conditions (n.s., *P* = 0.29; χ^2^ test). Pie chart color scheme as in Fig. 7A.

In the course of our experiments, we realized that there was an increased likelihood of no gastric mill rhythm occurring during incubation with any of our three experimental conditions (1 μM-UH, 1 μM-FH, or 10 μM-S) when it followed an incubation with any one of these conditions. To quantify this desensitization-like effect, we performed a set of experiments where the first incubation was any one of these conditions and the second incubation was 1 μM-UH (designated below as 1 μM-UH-D [D = desensitized]) (Fig. 11).

We first established that the distribution of the three outcomes (gastric mill rhythm, irregular gastric mill rhythm, no gastric mill rhythm) was distinct, and exhibited a large effect size, between 1 μM-UH and 1 μM-UH-D when the latter incubation followed any of our experimental conditions (Fig. 11A). This included a substantial increase in the likelihood of “no gastric mill rhythm” and decrease in the other two possible outcomes with 1 μM-UH-D. Furthermore, in contrast to the distinct distribution of outcomes between 1 μM-S and 1 μM-UH (Fig. 7A and Fig. 11A), there was no difference between 1 μM-S and 1 μM-UH-D (*P* = 0.34, χ^2^ test) (Fig. 11A). Similarly, the odds of gastric mill rhythm occurrence were distinct between 1 μM-UH and 1 μM-UH-D (odds ratios: 23.9, 95% CI 6.2: 138.8) but comparable for 1 μM-UH-D and 1 μM-S (odds ratio: 2.1, 95% CI 0.5: 12.5). Thus, there was a desensitizing action that suppressed the enhancing effect of hemolymph or elevated G-SIFamide concentration without suppressing the baseline effect of 1 μM-S.

We then established that each of our three experimental conditions (1 μM-UH, 1 μM-FH, or 10 μM-S), but not 1 μM-S, comparably desensitized the gastric mill circuit response to a subsequent incubation with 1 μM-UH (i.e., 1 μM-UH-D). Specifically, in each case the outcome distribution was distinct, and exhibited a large effect size, between 1 μM-UH-D and the other three conditions when the latter were not preceded by a desensitizing incubation (e.g., 1 μM-UH: *P* = 6 × 10^−16^, χ^2^ test, Cramer V = 0.7; Fig. 11B).

There were also no differences across the conditions used for the first incubation regarding the outcome in 1 μM-UH-D for either the number of long IC bursts (Incubation-1: 10 μM-S: 7.8 ± 5.2, *n* = 8; 1 μM-UH: 8.2± 7.2, *n* = 5; 1 μM-FH: 19.5 ± 9.9, *n* = 8; *P* = 0.94, Kruskal–Wallis test on ranks) or the number of pyloric rhythm interruptions (Incubation-1: 10 μM-S: 3.1 ± 2.8, *n* = 8; 1 μM-UH: 1.0 ± 0.8, *n* = 5; 1 μM-FH: 7.8 ± 4.6, *n* = 8; *P* = 0.78, Kruskal–Wallis test on ranks) across our 900-s incubation. In ~55% of these 1 μM-UH-D incubations, there were no long IC bursts or pyloric rhythm interruptions (Incubation-1: 1 μM-UH, *n* = 3/5; 1 μM-FH, *n* = 5/8; 10 μM-S, *n* = 4/8), whereas in the remaining nine experiments there was a range of values (e.g., no. of long IC bursts: ≤15, *n* = 4; ≥30, *n* = 5). However, in all nine of these latter experiments the IC response in the 1 μM-UH-D incubations was weakened (e.g., fewer >1 s bursts, briefer burst durations, less impact on pyloric cycle period), suggesting at least partial desensitization. These results suggest that there was a comparable level of desensitization across all three incubation conditions. The desensitization also did not reverse with a prolonged washout (2–6 h, *n* = 4).

This desensitizing response did not extend to all gastric mill circuit neurons. For example, despite the absence of a gastric mill rhythm, independent rhythmic bursting was elicited in the DG neuron during the 1 μM-UH-D incubations when any of the three conditions were used for the first incubation (1 μM-UH: *n* = 3/5 irregular bursting, *n* = 2/5 regular bursting; 1 μM-FH: *n* = 1/7 irregular bursting, *n* = 3/7 regular bursting, *n* = 3/7 no bursting; 10 μM-S: *n* = 3/8 irregular bursting, 3/8 regular bursting, 2/8 no bursting). In addition, relative to the slow pyloric rhythm in UH alone (Fig. 3), there was a comparable decrease in the pyloric cycle period by 1 μM-UH-D and 1 μM-UH (1 μM-UH: 1.2 ± 0.1 s, *n* = 5; 1 μM-UH-D 1.6 ± 0.1 s, *n* = 5; *P* = 0.13, Wilcoxon signed-rank test). There was no difference in the 1 μM-UH-D pyloric cycle period when any of the three conditions were used for the first incubation (1 μM-UH-D cycle period after: 10 μM-S, 2.0 ± 0.2 s; 1 μM-UH, 1.6 ± 0.1 s; 1 μM-FH, 1.8 ± 0.24 s; *P* = 0.5; ANOVA). Thus, the desensitizing effect of incubating 1 μM G-SIFamide with either of the two hemolymphs or 10 μM G-SIFamide in saline compromised activation of the gastric mill circuit without reducing or eliminating the decrease in the pyloric rhythm cycle period.

### G-SIFamide Is Below Detectable Limits in Unfed and Fed Hemolymph

Because the hemolymph did not elicit novel gastric mill circuit responses to G-SIFamide and its influence appeared comparable with a higher G-SIFamide concentration in saline, we used both LC-MS and MALDI-MS to test the hypothesis that the hemolymph was a source of additional G-SIFamide. Separate, pooled hemolymph samples from unfed crabs (*n* = 4) and fed crabs (*n* = 4) were each subjected to LC-MS analysis. No G-SIFamide, its degradants, or any SIFamide-like peptides [e.g., Val^1^-SIFamide; (92)] were identified in any of these samples, despite the identification of numerous other neuropeptides (Supplemental Table S1). Interestingly, although no SIFamide was identified in these samples, a SIFamide precursor-related protein (SIFamide PRP) was detected in both unfed and fed hemolymph (Supplemental Table S1).

**Figure 12.**
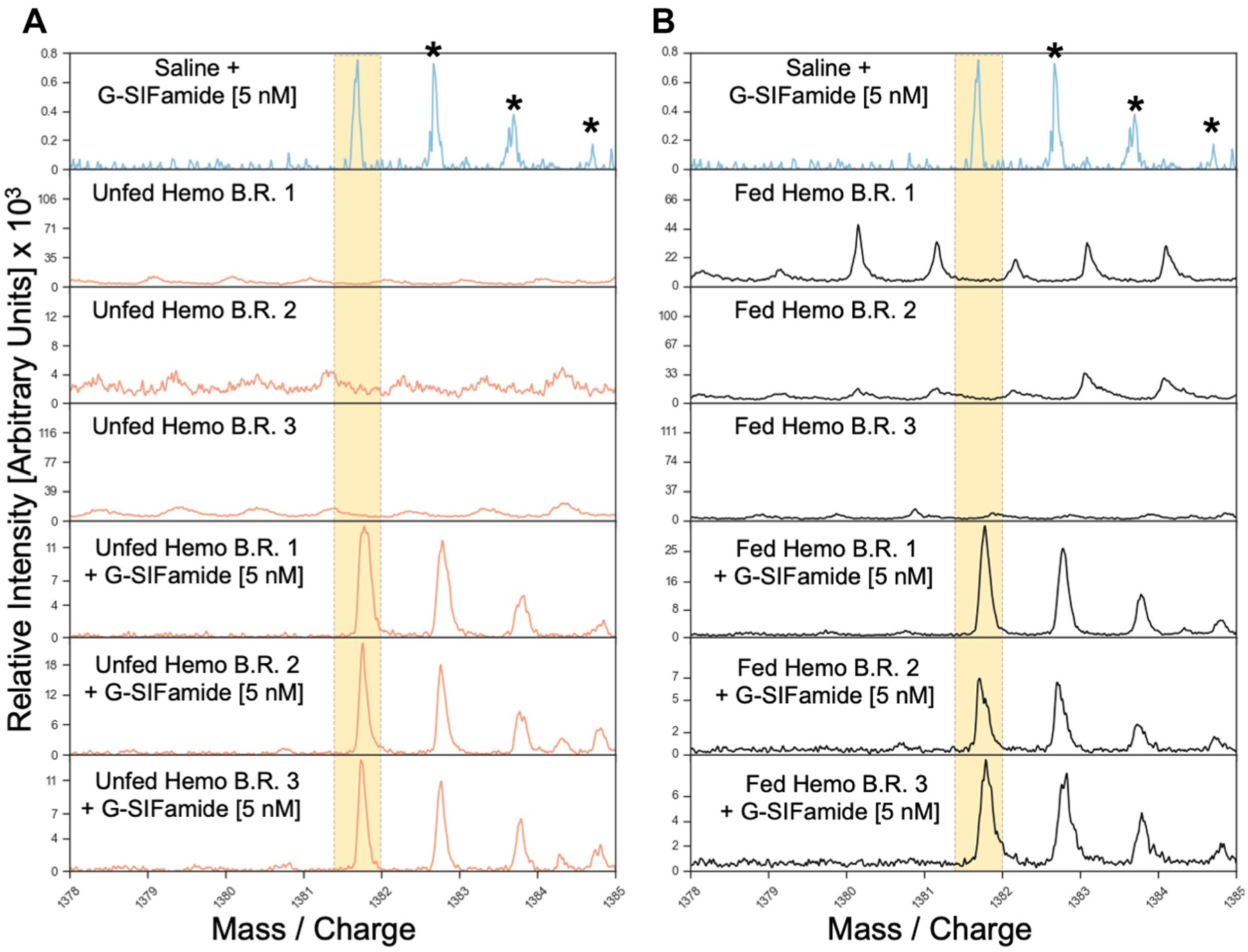
G-SIFamide is below detectable limits in unfed and fed hemolymph examined by MALDI mass spectrometry. *A*: the isotopic envelope of 5 nM G-SIFamide peptide standard added to saline (*top*) and unfed hemolymph from three different crabs (bottom three spectra). Spectra two through four are from unfed hemolymph samples from the same three crabs without the addition of G-SIFamide peptide standard. Yellow highlight indicates the singly charged, monoisotopic G-SIFamide peak (*m/z* 1381.74), with the subsequent isotopic peaks (*) representing the remaining aspects of its isotopic distribution. B.R., biological replicate. *B*: same as *A*, but with fed hemolymph.

To validate the absence of G-SIFamide in our LC-MS analyses, MALDI-MS analysis was performed on samples with and without synthetic G-SIFamide (5 nM) added to each sample after hemolymph removal but before peptide extraction (see materials and methods). As shown in Fig. 12, no endogenous G-SIFamide peak was detected in the MALDI-MS analysis of either unfed or fed hemolymph in samples without the addition of synthetic G-SIFamide, despite its detection in samples containing the added peptide. In both panels, the top spectrum represents the singly charged monoisotopic ion peak of synthetic G-SIFamide (5 nM; at *m/z* 1,381.74), along with its isotopic cluster peaks, serving as a reference for peak identification in the hemolymph samples. The next three spectra in both panels, from three separate unfed (Fig. 12A) or fed crabs (Fig. 12B), display no corresponding peaks at the G-SIFamide mass/charge (*m/z*) values. In contrast, these peaks are evident in the lower three spectra from hemolymph samples to which 5 nM synthetic G-SIFamide was added, confirming the sensitivity of our MALDI-MS approach. Notably, the G-SIFamide signal remained detectable throughout the sample preparation process, suggesting that the peptide is not substantially degraded under these sample preparation conditions, even at low concentrations. Collectively, these results indicate that G-SIFamide was not present at detectable levels in unfed or fed hemolymph under these conditions.

## DISCUSSION

We have established that the presence of *C. borealis* hemolymph increases the likelihood of gastric mill rhythm activation by the neuropeptide Gly^1^-SIFamide (G-SIFamide) relative to the same G-SIFamide concentration applied in saline. In addition, there was a behavioral state-specific distinction between the influence of unfed versus 1-h postfed hemolymph, with the gastric mill rhythm occurring more frequently with fed hemolymph, as might be expected given that the presence of food in the stomach activates regular chewing movements (93, 94). This latter outcome suggests that the hormone(s) responsible for this effect are present at a higher concentration after feeding. Alternatively, the increased effectiveness in fed hemolymph could result from a decrease in the presence and/or concentration of one or more co-circulating inhibitory hormones, of which there are several peptide families such as the allatostatins (16, 95–97).

The influence of 1 μM G-SIFamide in unfed or fed hemolymph on the gastric mill circuit was similar to that of 10 μM G-SIFamide in saline. For example, the frequency of occurrence of the gastric mill rhythm was considerably higher for these three conditions than with 1 μM-S. This effect was comparable between 1 μM-UH and 10 μM-Saline, whereas 1 μM-FH was even more effective in eliciting the gastric mill rhythm. In addition, although there were quantitative differences in some gastric mill activity parameters across the three experimental conditions (1 μM-UH, 1 μM-FH, 10 μM-S), the basic features of the gastric mill rhythm phase relationships were preserved. These outcomes suggest that the hormone(s) in unfed hemolymph boost the effectiveness of applied G-SIFamide so that it is comparable with a 10-fold higher peptide concentration in saline with respect to gastric mill rhythm activation but that a more complex set of actions, perhaps resulting partly from a different hormone balance in unfed and fed hemolymph, underlie the distinct activity level of the component neurons.

It remains to be determined what hormone(s) are responsible for the hemolymph influence on G-SIFamide activation of the gastric mill rhythm. One candidate derives from work in the spiny lobster *Panulirus interruptus*, where a CCK-like peptide present in the hemolymph activates the gastric mill rhythm in vivo and in the isolated STG (98–100). A previous mass spectrometry analysis identified the arthropod CCK ortholog, sulfakinin, in the *C. borealis* hemolymph, with comparable levels in unfed and 1-h postfed hemolymph (16). However, neither unfed nor fed hemolymph alone activated the gastric mill rhythm in *C. borealis*. The possibility that the hemolymph action on the G-SIFamide gastric mill rhythm is peptide(s)-mediated is reasonable, though, given that numerous neuropeptide hormones modulate the STG rhythms in *C. borealis* (16, 25, 32, 43). However, hormone-level actions of dopamine and serotonin also modulate the crab and lobster pyloric rhythm (42, 45, 101), and dopamine, serotonin, octopamine, GABA, and at least several steroid hormones are also present in the decapod crustacean hemolymph (102–109). It also remains possible that, despite being below detectable limits in the *C. borealis* hemolymph, a low G-SIFamide concentration therein, acting via a distinct high-affinity receptor/mechanism, could influence the gastric mill circuit response to neuronal release-levels of the peptide, as is the case for dopamine actions on the pyloric rhythm in *P. interruptus* (44, 45).

The microcircuit and associated behavioral response to what are often arbitrarily selected, hormone-like concentrations of some individual amine (45, 110), peptide (2, 9), and steroid hormones (4, 6, 12, 22) has been studied using in vitro and/or in vivo preparations in several systems. In some cases, these responses have been observed in different behavioral states, such as feeding and metabolic state (12), seasonal/reproductive state (4, 7), and stress (6). As in the present study, however, it remains to be determined whether these hormone actions required the parallel influence of co-circulating hormones.

### Candidate Mechanisms Underlying the Enhanced G-SIFamide Action

There were no novel gastric mill neuron responses to G-SIFamide in the presence of hemolymph relative to applying G-SIFamide in saline (50, 51, 83, 111–113). This result suggests that one or more non-SIFamide hormones act by strengthening the influence of the applied peptide. This suggestion was supported by three additional observations. First, the likelihood of gastric mill rhythm occurrence was comparable in the 1 μM-UH and 10 μM-S conditions. Second, the hemolymph was not providing substantial, if any, additional G-SIFamide, because no SIFamide peptide family members were detected in our mass spectrometry analysis of unfed and fed hemolymph. The presence of a G-SIFamide PRP, but no G-SIFamide, in our LC-MS analysis suggests that either G-SIFamide was released into the hemolymph but subsequently degraded before the hemolymph reached our collection site, or the PRP and G-SIFamide are not coreleased. Third, incubation with 1 μM-UH, 1 μM-FH, or 10 μM-S, but not 1 μM-S, had comparable desensitizing effects on the gastric mill circuit response to subsequent G-SIFamide applications.

There are a number of candidate mechanisms by which hormonal strengthening of the G-SIFamide actions might occur. For example, one or more hormones might activate the same ionic current(s) in the same STG neurons as G-SIFamide. There is precedent for this possibility in that there is convergent activation of the same, voltage-dependent inward current by several different neuropeptides, and a muscarinic agonist, in STG neurons (27, 114, 115). Such convergence, albeit onto different ionic currents, also occurs in other systems (116–118). The likelihood of this possibility, however, is diminished by the observation that the hemolymph concentration of peptide hormones is typically low (≤1 nM), even under behavior-enriching conditions in decapod crustaceans (119–123). Such low concentrations would not likely be impactful for summing with the applied G-SIFamide (1 μM) to increase the amplitude of the G-SIFamide activated current(s) (111, 114, 115).

Alternatively, one or more hormones may out-compete G-SIFamide for binding to an extracellular peptidase that cleaves and inactivates G-SIFamide within the STG neuropil. As occurs in all nervous systems (124–126), extracellular peptidase activity limits the actions of at least some neuropeptides in the STG (127–129). Many, and perhaps all, extracellular peptidases bind and cleave at least several unrelated neuropeptides, due to their selectivity for particular peptide bonds that are often present in unrelated peptides (124, 125). Another possibility is that one or more hemolymph hormones trigger the insertion of additional SIFamide receptors from intracellular compartments. Neuropeptide receptors are often present in endosomes, from which location they can be trafficked and inserted into the plasma membrane (130, 131).

The hemolymph effect may instead result from one or more hormones causing an increased neuropil concentration of G-SIFamide by eliciting its release from the STG terminals of SIFamidergic projection neurons. The STG terminals of projection neurons, including the G-SIFamidergic neuron MCN5, are responsive to local synaptic input and applied modulators (50, 132–136). There are two pairs of G-SIFamide containing projection neurons that innervate the STG, and stimulating at least one of them (MCN5) elicits a G-SIFamidergic gastric mill rhythm similar to G-SIFamide application (50, 83).

### Circuit-Specific Desensitization

The desensitization response of the gastric mill circuit may be a consequence of our incubation protocol, because repeated 5 μM G-SIFamide superfusion does not elicit this response (50, 51, 83, 112, 113). Nevertheless, its value in the current context rests on its comparable frequency of occurrence after applying 1 μM-UH, 1 μM-FH, or 10 μM-S, but not after applying 1 μM-S. As discussed earlier, this outcome supports the hypothesis that there is a shared mechanism underlying the gastric mill circuit response to 1 μM G-SIFamide in both types of hemolymph and 10 μM G-SIFamide in saline.

The lack of a gastric mill circuit desensitizing response in previous studies to repeatedly superfusing 5 μM G-SIFamide presumably results from the different methodology. The difference between superfusion and incubation may result from only the outer layer of the STG neuropil being subjected to the full concentration of the superfused peptide, with a lower concentration penetrating deeper, whereas the incubated peptide concentration more effectively penetrates the neuropil.

The fact that G-SIFamide still strengthened the pyloric rhythm during the gastric mill-desensitization protocol suggests the presence of more than one SIFamide receptor type, with distinct desensitization properties, in at least some STG neurons. This includes the possibility of different SIFamide receptors being co-present in the gastro-pyloric neurons such as IC, where only their gastric mill-related response is desensitized. Distinct, desensitization-specific receptor types (e.g., tachykinin receptors) occur in other systems (137, 138). The possible presence of distinct SIFamide receptors in STG neurons is supported by recent work in another arthropod where two different G-SIFamide receptors were identified (139).

Peptide receptor desensitization often results from receptor internalization following ligand-receptor binding, subsequent receptor phosphorylation and arrestin-mediated sequestration into endosomes (130, 131, 137, 140–143). This process can result from either homologous desensitization, for example triggered by G-SIFamide binding to its receptor (e.g., by 10 μM G-SIFamide), or heterologous desensitization due to activation of a distinct GPCR (e.g., by a hemolymph hormone). The internalized receptors are eventually trafficked to lysosomes for degradation or recycled to the plasma membrane. The fact that the desensitized gastric mill response to G-SIFamide did not recover for at least 6 h suggests that in these experiments a G-SIFamide receptor was internalized and targeted for degradation. The lack of a decline in the gastric mill rhythm during the initial 15 min G-SIFamide incubation with hemolymph or at an elevated peptide concentration in saline, but no rhythm occurring in the subsequent incubation 1 h later, suggests that receptor internalization is a slow process or it happens relatively quickly but the subsequent metabotropic action persists.

### Circuit State-Specific Hemolymph Influence

In reduced, in vitro preparations, applying an individual neuromodulator or activating a modulatory neuron can differently alter different states of the same circuit (26, 29, 44, 144–148). We show here that this outcome also occurs in the isolated stomatogastric system under the influence of a complete, naturally occurring population of hormones. Specifically, there were different feeding state-specific actions of hemolymph on the G-SIFamide-gastric mill rhythm and the previously studied VCN-triggered gastric mill rhythm in *C. borealis* (19). In the latter study, only fed hemolymph altered the VCN-rhythm, and that influence was distinct from the impact of fed hemolymph on the G-SIFamide-rhythm. For example, during the VCN-rhythm the most prominent protractor phase neuron was LG, and there were no prolonged (>1 s) IC bursts or pyloric rhythm interruptions (19). It remains to be determined whether these distinct circuit responses result from the influence of different hemolymph hormones or the same hormone(s) influencing different circuit states.

In summary, we found that providing a feeding-related neural circuit in the isolated nervous system with the complete population of naturally occurring hormones from two different feeding states enhanced a neuropeptide activation of that circuit in a feeding state-specific manner. The hormone(s) responsible for this action remain to be identified, but it is unlikely due to the activating neuropeptide (G-SIFamide), as it was below detectable limits (<5 nM) in the hemolymph. These results suggest that circulating hormones can facilitate motor pattern activation, even when they do not elicit said activation themselves.

## Supplementary Material

Supplemental Table 1

Figure S2

Figure S1

Figure S3

Supplemental Figs. S1–S3 and Supplemental Table S1: https://doi.org/10.6084/m9.figshare.29260520.

## Figures and Tables

**Table 1.** Gastric mill rhythm (IC neuron) and pyloric rhythm (PD neuron) burst parameters during different G-SIFamide incubation conditions

Parameter	Means ± SE			95% Confidence Interval from Difference of Means				Effect Size (Cohen’s *d*)		
	1 μM-FH *n* = 38, 11	1 μM-UH *n* = 23, 7	10 μM-S *n* = 42, 12	1 μM-FH vs. 1 μM-UH	1 μM-FH vs. 10 μM-S	1 μM-UH vs. 10 μM-S		1 μM-FH vs. 1 μM-UH	1 μM-FH vs. 10 μM-S	1 μM-UH vs. 10 μM-S
No. of IC spikes per long (>1 s) burst	72.1 ± 6.9	61.4 ± 6.7	45.0 ± 4.9	0.6:20.6; *P* = n.s.	18.4:36.0; *P* < 0.00001		7.7:25.3; *P* < 0.0001	N/A	1.2	1
GMR-related IC spike freq., Hz	27.6 ± 1.7	21.3 ± 1.1	21.0 ± 1.4	4.2:8.4; *P* < 0.00001	4.2:9.0; *P* < 0.00001		−1.3:2.1; *P* = n.s.	1.3	1.2	N/A
GMR-related IC duty cycle, %	19.0 ± 1.8	15.2 ± 1.4	15.1 ± 1.1	1.7:6.0; *P* < 0.001	1.8:6.1; *P* < 0.001		−1.9:2.0; *P* = n.s.	0.6	0.6	N/A
GMR-related IC cycle period, s	13.9 ± 0.7	18.3 ± 1.6	14.2 ± 0.7	−6.1:−2.6; *P* < 0.00001	−1.5:1.0; *P* = n.s.		2.1:6.0; *P* < 0.001	−1.5	N/A	1.5
No. of GMR-related IC bursts	15.0 ± 0.7	12.0 ± 1.1	15.4 ± 1.0	1.7:4.3; *P* < 0.00001	−1.9:1.0; *P* = n.s.		−5.0:−1.8; *P* < 0.0001	0.9	N/A	−0.9
GMR-related IC burst duration, s	2.6 ± 0.2	2.7 ± 0.3	2.1 ± 0.1	−0.5:0.2; *P* = n.s.	0.3:0.8; *P* < 0.00001		0.4:1.0; *P* < 0.00001	N/A	0.8	1.2
No. of PR cycles > 150% control	13.1 ± 1.4	11.0 ± 1.2	10.4 ± 1.1	0.5:3.7; *P* < 0.01	0.9:4.4; *P* < 0.01		−1.3:2.5; *P* = n.s.	0.4	0.4	N/A
PR cycle per. (s) control (<10 IC spikes)	1.1 ± 0.1	1.3 ± 0.1	1.1 ± 0.1	−0.3:−0.1; *P* < 0.00001	−0.1:0.1; *P* = n.s.		0.1:0.3; *P* < 0.00001	−1.4	N/A	1.5
Mean duration (s) of PR delay	2.4 ± 0.1	2.9 ± 0.3	2.3 ± 0.2	−0.8:−0.2; *P* < 0.001	−0.2:0.3; *P* = n.s.		0.3:0.9; *P* < 0.0001	−1.1	N/A	1.2
Mean PR delay (% control)	218.9 ± 11.6	223.5 ± 16.5	215.9 ± 15.6	−26.2:18.0; *P* = n.s.	−18.0:24.7; *P* = n.s.		−18.3:32.7; *P* = n.s.	N/A	N/A	N/A

*n* = *X, Y: X* = number of segments, *Y* = number of stomatogastric ganglia (STGs). Statistics: Bootstrapped confidence interval of mean. Effect size calculated using unbiased Cohen’s *d*, magnitude (+ or −): Small, 0.2–0.5; Medium, 0.5–0.8; Large, ≥0.8. FH, fed hemolymph; GMR, gastric mill rhythm; IC, inferior cardiac; PD, pyloric dilator; PR, pyloric rhythm; S, saline; UH, unfed hemolymph.

**Table 2.** Burst parameters of gastric mill neurons LG, DG, and AM during the G-SIFamide gastric mill rhythm

Parameter	Means ± SE			95% Confidence Interval from Difference of Means			Effect Size (Cohen’s *d*)		
	1 μM-FH *n* = 38, 11	1 μM-UH *n* = 23, 7	10 μM-S *n* = 42,12	1 μM-FH vs. 1 μM-UH	1 μM-FH vs. 10 μM-S	1 μM-UH vs. 10 μM-S	1 μM-FH vs. 1 μM-UH	1 μM-FH vs. 10 μM-S	1 μM-UH vs. 10 μM-S
No. of LG spikes per burst	14.7 ± 3.1	7.2 ± 1.5	19.3 ± 2.6	3.4:11.3; *P* < 0.001	−9.4:0.0; *P* = n.s.	−15.6:−8.5; *P* < 0.00001	1	N/A	−1.6
LG spike freq., Hz	4.5 ± 0.5	2.7 ± 0.4	4.9 ± 0.4	1.2:2.5; *P* < 0.00001	−1.1:0.4; *P* = n.s.	−2.8:−1.6; *P* < 0.00001	1.4	N/A	−1.7
LG burst duration, s	3.4 ± 0.8	2.6 ± 0.6	4.5 ± 0.7	−0.2:1.8; *P* = n.s.	−2.2:0.1; *P* = n.s.	−2.9:−0.8; *P* < 0.01	N/A	N/A	−0.8
No. of DG spikes per burst	142.1 ± 52.7	134.4 ± 25.3	183.7 ± 23.8	−42.4:1.5; *P* = n.s.	−95.6:11.6; *P* = n.s.	−91.0:−0.4; *P* = n.s.	0.3	N/A	N/A
DG spike freq., Hz	15.4 ± 1.2	12.6 ± 1.5	13.7 ± 0.7	0.9:4.6; *P*< 0.01	0.2:3.2; *P* = n.s.	−2.6:0.5; *P* = n.s.	0.8	N/A	N/A
DG burst duration, s	8.8 ± 2.5	10.0 ± 1.4	13.8 ± 1.7	−3.9:1.1; *P* = n.s.	−8.3:−1.4; *P* < 0.01	−6.7:−0.4; *P* = n.s.	N/A	−0.5	N/A
No. of AM spikes per burst	25.6 ± 4.9	189.3 ± 364.3	82.2 ± 28.6	−331.3:132.9; *P* = n.s.	−80.2:−29.1; *P* < 0.00001	−191.4:279.3; *P* = n.s.	N/A	−1	N/A
AM spike freq., Hz	7.7 ± 0.4	6.7 ± 0.7	8.6 ± 0.7	0.2:1.8; *P* = n.s.	−1.6:−0.1; *P* = n.s.	−2.8:−1.0; *P* < 0.00001	N/A	N/A	−0.9
AM burst duration, s	2.6 ± 0.5	32.3 ± 58.2	9.5 ± 6.7	−59.0:17.6; *P* = n.s.	−10.4:−1.6; *P* = n.s.	−27.0:53.1; *P* = n.s.	N/A	N/A	N/A

*n* = *X, Y: X* = number of segments, *Y* = number of stomatogastric ganglia (STGs). Statistics: Bootstrapped confidence interval of mean. Effect size calculated using unbiased Cohen’s *d*, magnitude (+ or −): Small, 0.2–0.5; Medium, 0.5–0.8; Large, ≥0.8. AM, anterior median; DG, dorsal gastric; FH, fed hemolymph; GMR, gastric mill rhythm; IC, inferior cardiac; LG, lateral gastric; PD, pyloric dilator; PR, pyloric rhythm; S, saline; UH, unfed hemolymph.

## Data Availability

A module containing functions used for analysis of electrophysiology data are available at https://github.com/LoganJF/hormonaltuning, and data are available at (https://github.com/LoganJF/hormonaltuning/tree/main/hormonaltuning/examples) or upon request from M.P.N. Li laboratory crustacean peptide database is available at: https://www.lilabs.org/resources or upon request from L.L.
