# Supplementary figures and images for: Feeding state-specific hormonal tuning of neural circuit modulation
Source: J Neurophysiol. Author manuscript; Available in PMC 2025 Sep 20. (PMC12450063; doi:10.1152/jn.00164.2025)

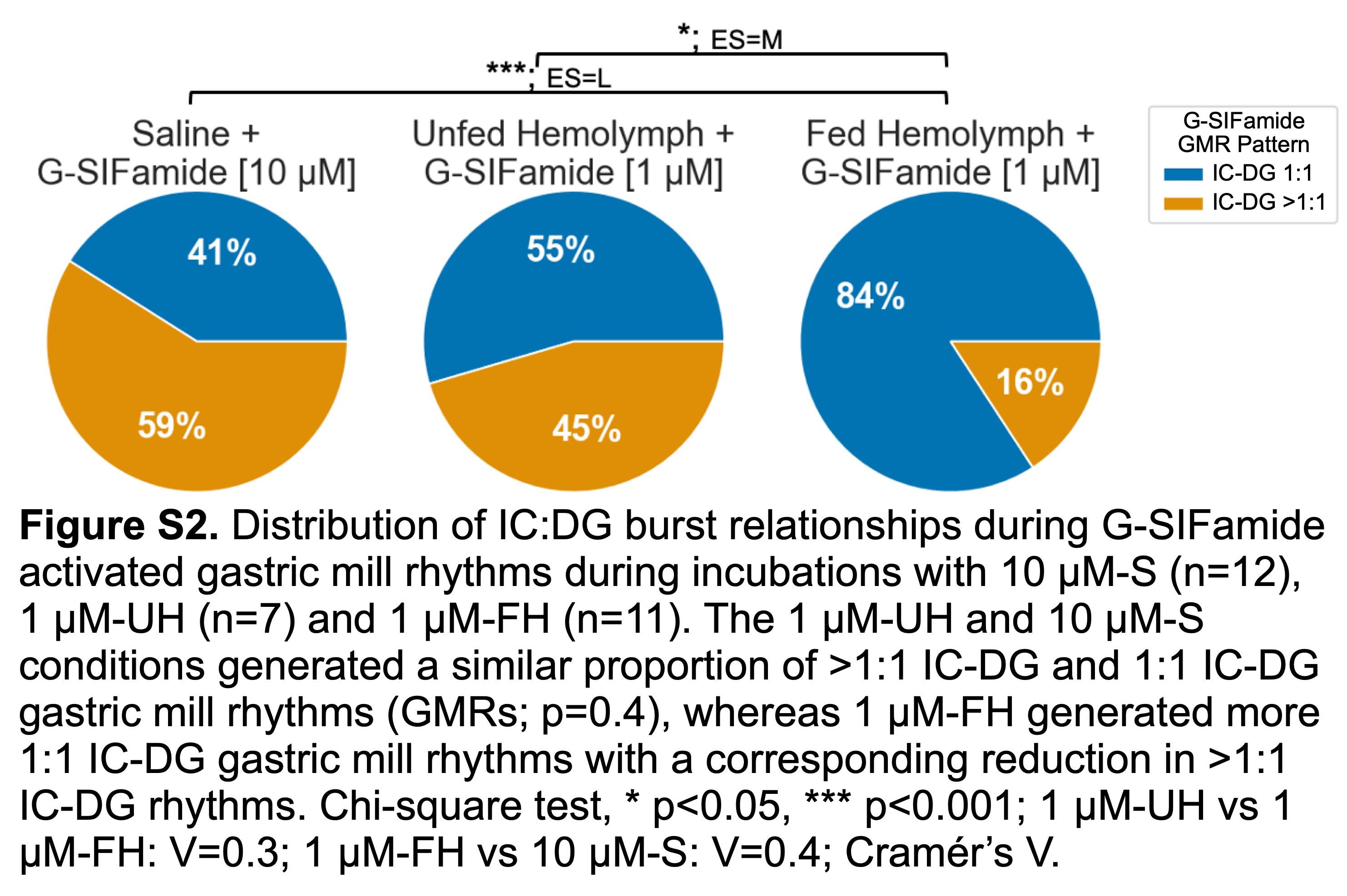

Supplement: Figure S2 [file NIHMS2111039-supplement-Figure_S2.tiff]

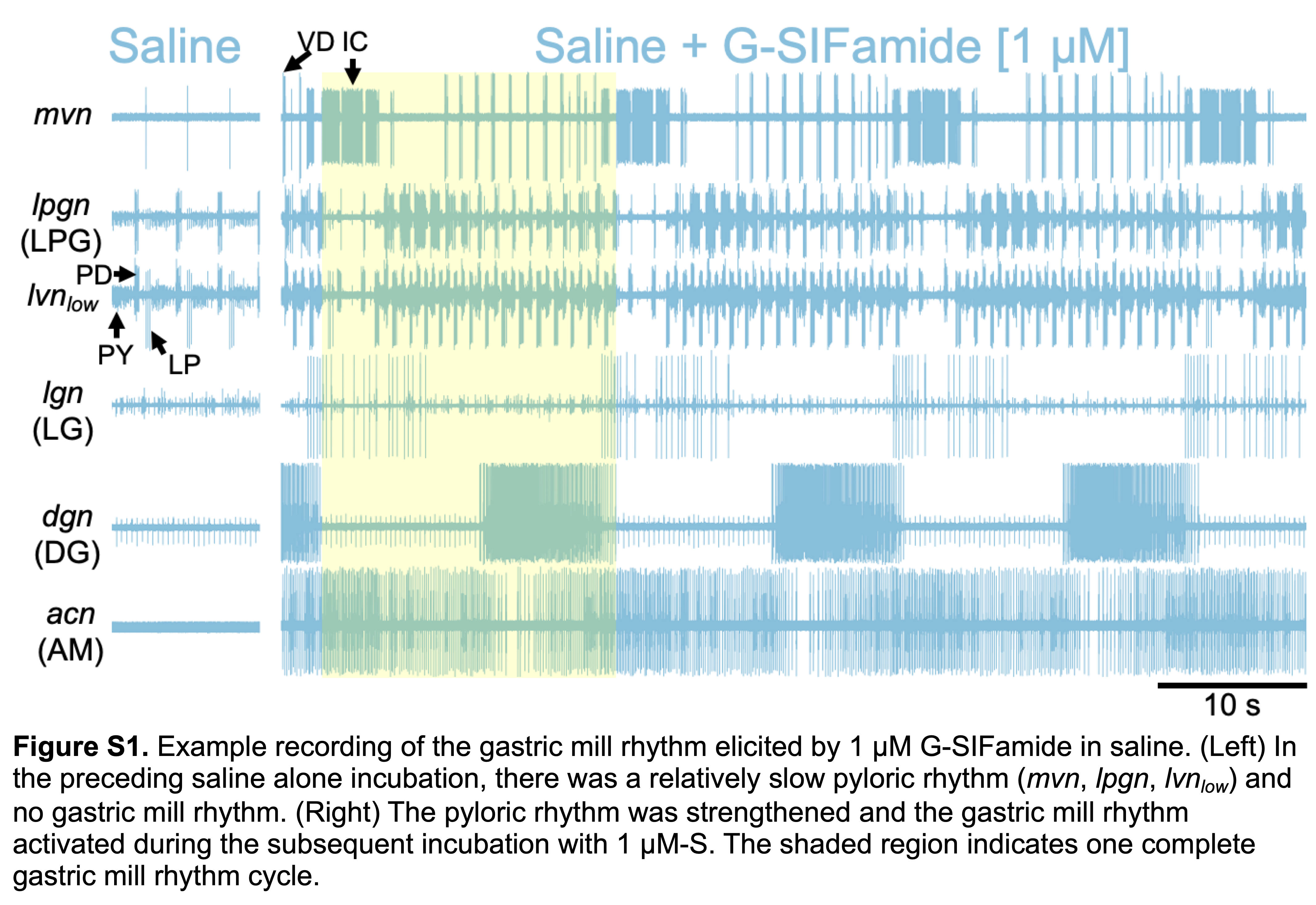

Supplement: Figure S1 [file NIHMS2111039-supplement-Figure_S1.tiff]

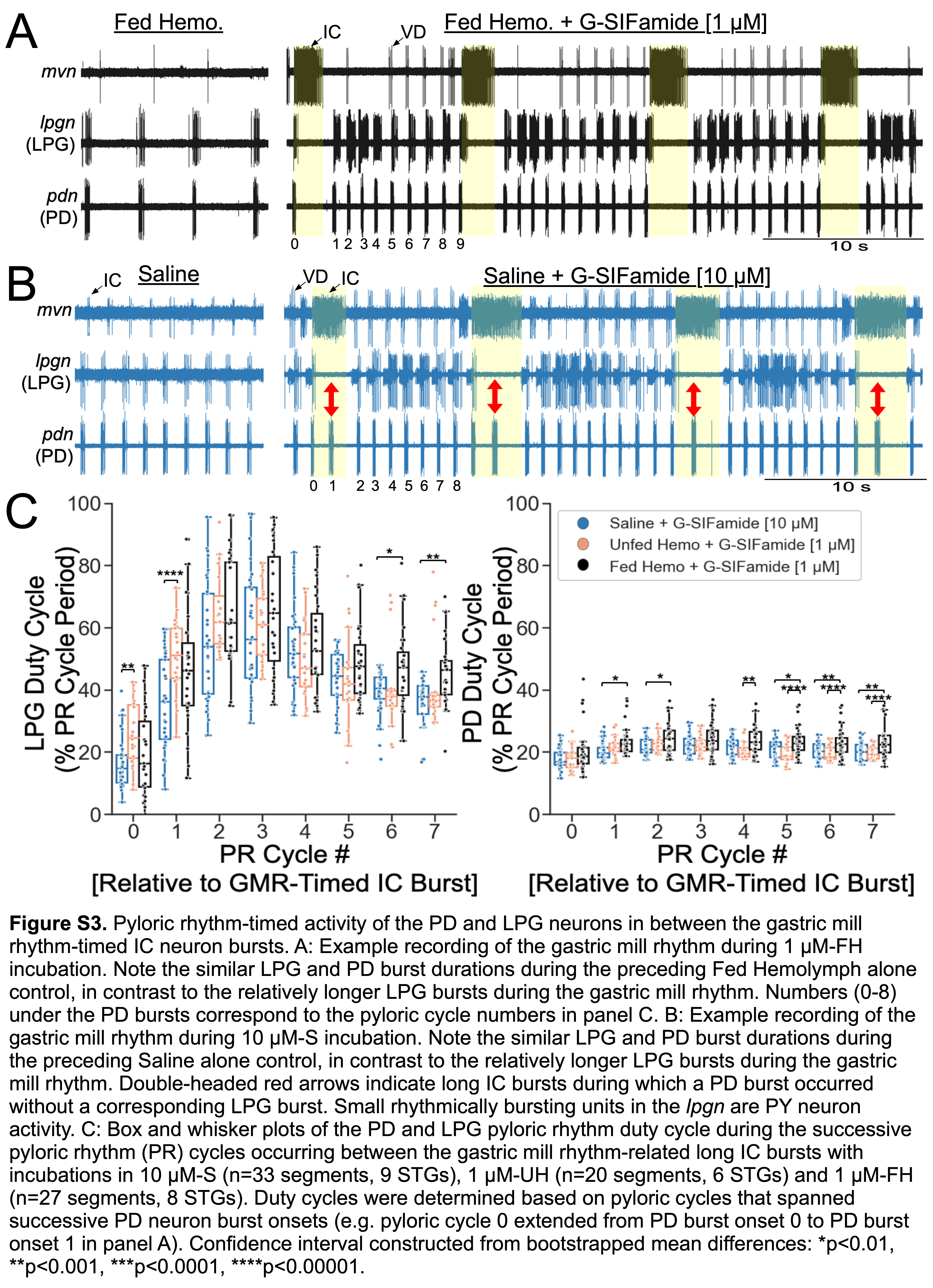

Supplement: Figure S3 [file NIHMS2111039-supplement-Figure_S3.tiff]
